# Development and proof-of-concept demonstration of a clinical metagenomics method for the rapid detection of bloodstream infection

**DOI:** 10.1186/s12920-024-01835-5

**Published:** 2024-03-05

**Authors:** Lluis Moragues-Solanas, Thanh Le-Viet, Elinor McSorley, Carl Halford, Daniel S. Lockhart, Alp Aydin, Gemma L. Kay, Ngozi Elumogo, William Mullen, Justin O’Grady, Matthew W. Gilmour

**Affiliations:** 1 Quadram Institute Bioscience, Norwich Research Park, Rosalind Franklin Road, Norwich, Norfolk, NR4 7UQ UK; 2 University of East Anglia, Norwich, Norfolk, UK; 3 Momentum Bioscience Ltd, Blenheim Office Park, Witney, Oxfordshire UK; 4 Defence Science and Technology Laboratory, Porton Down, Salisbury, Wiltshire UK; 5 Norfolk and Norwich University Hospital, Colney Lane, Norwich, UK; 6 Oxford Nanopore Technologies Plc, Quadram Institute Bioscience, Norwich, UK

**Keywords:** Clinical diagnostics, Metagenomics, Bacteraemia, Sepsis, Bacterial infection, Long-read sequencing

## Abstract

**Background:**

The timely and accurate diagnosis of bloodstream infection (BSI) is critical for patient management. With longstanding challenges for routine blood culture, metagenomics is a promising approach to rapidly provide sequence-based detection and characterisation of bloodborne bacteria. Long-read sequencing technologies have successfully supported the use of clinical metagenomics for syndromes such as respiratory illness, and modified approaches may address two requisite factors for metagenomics to be used as a BSI diagnostic: depletion of the high level of host DNA to then detect the low abundance of microbes in blood.

**Methods:**

Blood samples from healthy donors were spiked with different concentrations of four prevalent causative species of BSI. All samples were then subjected to a modified saponin-based host DNA depletion protocol and optimised DNA extraction, whole genome amplification and debranching steps in preparation for sequencing, followed by bioinformatical analyses. Two related variants of the protocol are presented: 1mL of blood processed without bacterial enrichment, and 5mL of blood processed following a rapid bacterial enrichment protocol—Sepsi*PURE*.

**Results:**

After first identifying that a large proportion of host mitochondrial DNA remained, the host depletion process was optimised by increasing saponin concentration to 3% and scaling the reaction to allow more sample volume. Compared to non-depleted controls, the 3% saponin-based depletion protocol reduced the presence of host chromosomal and mitochondrial DNA < 10^6^ and < 10^3^ fold respectively. When the modified depletion method was further combined with a rapid bacterial enrichment method (Sepsi*PURE*; with 5mL blood samples) the depletion of mitochondrial DNA improved by a further > 10X while also increasing detectable bacteria by > 10X. Parameters during DNA extraction, whole genome amplification and long-read sequencing were also adjusted, and subsequently amplicons were detected for each input bacterial species at each of the spiked concentrations, ranging from 50–100 colony forming units (CFU)/mL to 1–5 CFU/mL.

**Conclusion:**

In this proof-of-concept study, four prevalent BSI causative species were detected in under 12 h to species level (with antimicrobial resistance determinants) at concentrations relevant to clinical blood samples. The use of a rapid and precise metagenomic protocols has the potential to advance the diagnosis of BSI.

**Supplementary Information:**

The online version contains supplementary material available at 10.1186/s12920-024-01835-5.

## Background

Bloodstream infection (BSI) represent a significant global health problem, with an estimated 50 million cases per year and a 20% mortality rate [1]. Despite this high incidence and mortality, diagnosis of BSI remains challenging due to the low sensitivity and long turnaround time to results, where it is estimated that current gold standard blood culture (BC) methods can detect only 30–50% of the cases within the first 2 days after collection of blood samples for the majority of species, and as long as 5 days for other species [2–4]. Novel and rapid diagnostics are needed to support the identification of aetiologic agents of BSI and for the refinement of antibiotic treatment [5]. Antibiotics are critical for the treatment and rapid empiric administration of antibiotics is recommended upon suspicion of BSI [2]. Delays in diagnosis or the incorrect administration of antibiotics can lead to increased mortality [6, 7].

Culture-based BSI diagnostics has been improved through automated systems that enable higher throughput sample processing, continuous monitoring, or improved standardization [8]. Examples include matrix-assisted laser desorption ionization-time of flight mass spectrometry (MALDI-TOF MS), which quickly detect microbes to the species-level directly from positive BC specimens or from cultured isolates [9], and automated species identification and antimicrobial susceptibility testing (ID/AST), such as VITEK®2 cards which have substantially improved detection of antibiotic resistant organisms [10]. However, both methods still rely on positive blood culture (BC) [11]. The dependence on BC calls for an urgent response to find a rapid and more effective method to diagnose BSI [12, 13].

The field of Clinical Metagenomics (CMg) has recently emerged due to substantial technological sequencing advances that provide practical improvements in time-to-result and cost-per-sample, and it is now a viable diagnostic approach to consider sequencing of pathogens directly from human tissue samples [14, 15]. Direct sequencing from blood samples has the potential to overcome the issues associated with BC or nucleic acid amplification tests [16], yet whole blood samples present a challenge for development of CMg pipelines. The number of infecting microbial cells during adult BSI are normally < 10 CFU/mL [17] while host DNA contributed from white blood cells and other constituents of blood is in far excess of microbial DNA [18]. Therefore, extraction of sufficient detectable bacterial DNA is a challenge [19], where the metagenomic identification of bacterial pathogens is almost impossible without the use of efficient pathogen enrichment methods or extended sequencing times. Metagenomics samples can also contain a large amount of contaminant reads originating from multiple sources such as the environment, reagents or manual handling [20, 21], resulting in false positives and complications to the interpretation of metagenomics sequencing results, especially if common agents of BSI such as *E. coli* can also be found as contaminants [22]. To address some of the issues associated with the low number of microorganisms present in BSI samples, pre-analytical bacterial DNA enrichment steps such as Whole Genome Amplification (WGA) or host DNA depletion through differential chemical lysis (e.g. with saponin) or DNA amplification can be incorporated into CMg pipelines. However, these methods may indiscriminately enrich the presence of the contaminants and background DNA from different sources [21, 23]. Thus, no CMg BSI pipelines have been routinely implemented as a clinical diagnostic as they do not have the same sensitivity and specificity levels as the current gold standard diagnostics [24].

Recently, some CMg approaches using cell-free DNA (cfDNA) have been developed for the diagnosis of BSI [25, 26]. However, this approach is unable to detect antimicrobial resistance (AMR) genes due to the lack of whole genome coverage and is prone to false positive results caused by the detection of cfDNA from species that are not the causative agent of infection [24, 27–29]. CMg directly applied to whole blood can theoretically detect bacterial AMR genes [30, 31]. Despite this approach being highly successful when applied in other tissues, such as sputum for the detection of respiratory infections [32], direct applications in blood have been limited, with reduced sensitivity due to the high levels of host genetic material present [33–35]. To overcome the large abundance of host DNA in whole blood, most methods rely on selective host depletion steps that leave intact the microorganisms present [36, 37]. However, to date, host depletion methods have not been effective enough to allow rapid and accurate BSI diagnosis.

**Fig. 1:**
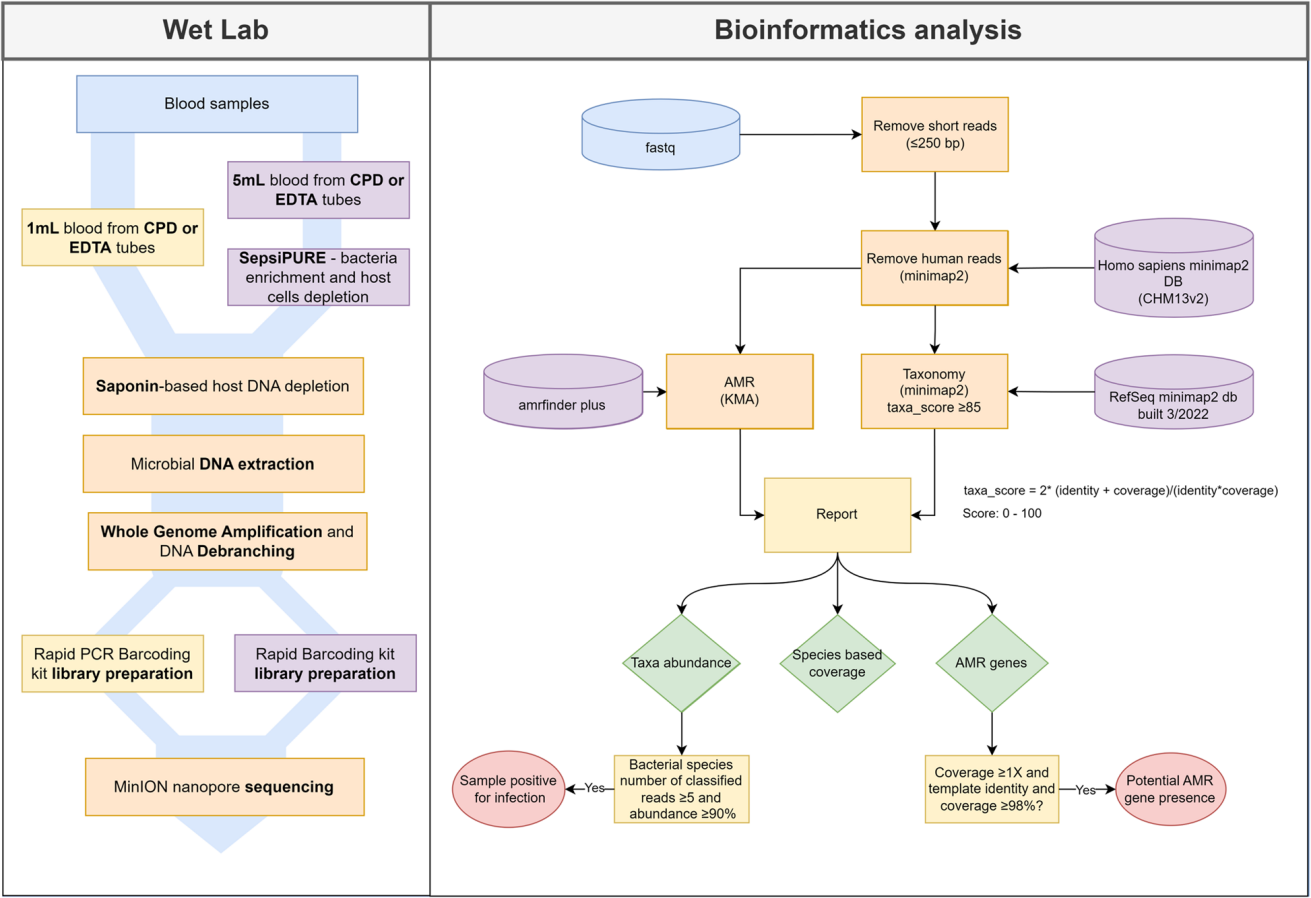
Schematic representation of steps involved in 1mL and 5mL CMg pipelines. Left panel: In triplicates, 50–100 CFU/mL, 5–10 CFU/mL, 1–5 CFU/mL and NTC of *E. coli* (CTX-M-15), *S. aureus*, *K. pneumoniae* or *E. faecalis* cultures were spiked into 1mL (Standard protocol) or 5mL (Quick-enrichment protocol) of whole blood samples in EDTA or CPD. 5mL samples underwent Sepsi*PURE* extraction and both 5mL and 1mL samples went through saponin-based host DNA depletion and subsequent DNA extraction. DNA extracts were whole genome amplified and debranched/digested. Samples were subjected to either Rapid PCR Barcoding (SQK-RPB004) or Rapid barcoding kit (SQK-RBK004) library preparation and nanopore sequenced (3 h) with Oxford Nanopore MinION. Right panel: Short (≤ 250bp) fastq were removed and then mapped against the human chromosome to eliminate remaining host DNA reads. Remaining bacterial reads were taxonomically classified using minimap2 and filtered according to their taxa_score and AMR determinants identified using KMA. Reports for taxonomic abundance, species coverage mapped metrics and AMR determinants detection data were produced. Samples were deemed positive for infection if bacterial species number of reads was ≥ 5 and relative abundance over the total number of reads ≥ 90%. For AMR gene detection, gene specific coverage had to be ≥ 1 and template identity and coverage ≥ 98%

Here we present a proof-of-concept study on a CMg method that accurately detects key bacterial species spiked in whole blood samples at clinically relevant concentrations. The method includes the following steps: host DNA depletion, DNA extraction, whole genome amplification, and bioinformatic analysis (Fig. 1). Prior to determining limits of detection of the method, key parameters of each step such as saponin concentration were optimised in the context of depleting host cells and/or recovering microbial DNA from blood samples, and additionally, an enrichment step was explored as an addendum to the initial steps of the method to improve analytical sensitivity for microbial DNA and efficiency of host depletion. Both arms of the protocol (i.e., the standard protocol without an enrichment step, versus the protocol with an enrichment addendum) utilise real-time nanopore long-read sequencing and results can be available approximately 9h and 12h, respectively, from sample to result. With further development using clinical specimens, these methodologies could be implemented for the diagnosis of BSI and improved management of sepsis.

## Methods

### Blood sample collection

Whole human blood samples were commercially acquired from Cambridge Bioscience. Blood samples were collected from healthy donors aged between 18–60 and were supplied in Ethylenediaminetetraacetic acid (EDTA) or Citrate–Phosphate-Dextrose (CPD) anticoagulants and stored under chilled (4˚C) conditions.

### Spiking blood samples and colony count

Different volumes of blood corresponding to variants of the core protocol (1mL, ‘*1mL standard protocol’;* or 5mL ‘*5mL quick-enrichment protocol’*) were spiked using serial dilutions (in PBS) of *Escherichia coli* (ATCC 10536 or NCTC 13441), *Staphylococcus aureus* (ATCC 25923), *Klebsiella pneumoniae* (ATCC 13882) and *Enterococcus faecalis* (NCTC 13779) from overnight cultures in Luria–Bertani (LB) broth or TRM media (Momentum Bioscience Ltd. (MBL)). For non-template control samples (NTC), blood samples were spiked with the same volume of PBS containing no bacterial species. NTC samples were subjected to the remainder of the full pipeline alongside the spiked samples. Overnight spiked concentrations were calculated by plating the dilutions on LB agar plates and growing them overnight to determine the CFU/mL spiked into the samples.

### Saponin host-depletion protocol optimisation

The standard host depletion protocol (saponin at 1% final concentration [38]) was compared to a variant protocol with an increased final concentration of saponin (3%). For both protocols, 200μL of spiked blood (50–100 CFU/mL *E. coli*) were combined with saponin (in PBS) at a final concentration of 1% or 3%, 200μL of HLSAN Buffer (in water 5.5M NaCl and 100mM MgCl2) and 10μL of HLSAN DNase (ArticZymes) in a 1.5mL Eppendorf tube. Samples were incubated at 37˚C for 10 min at 1000rpm on a ThermoMixer (Eppendorf). The depletion efficiency of the standard protocol was tested by sequencing saponin-depleted samples and comparing them to the same non-depleted controls. Sequencing results were analysed by taxonomically classifying the reads into human chromosome, human mitochondria and bacterial reads (see Bioinformatics). The standard 1% and variant 3% saponin protocol DNA was extracted and compared by qPCR by measuring the effects of these methods on both host DNA (chromosomal and mitochondrial DNA) and microbial DNA (qPCR targets in Additional File: Table S1). Finally, a scaled-up saponin-based depletion protocol, which allowed an increase in sample volume processed, was optimized and compared to the protocol described previously (saponin at 3% final concentration). The ‘scaled-up protocol’ was tested by spiking with 50–100 CFU/mL of *E. coli* into 1mL of blood and combined with saponin at a final concentration of 3%, 1mL of HLSAN Buffer and 10μL of HLSAN DNase on a 5.0mL Eppendorf tube. Samples were incubated at 37˚C for 30 min at 1000rpm on a ThermoMixer. Protocols were compared by qPCR by measuring the effects of these methods on both host DNA (chromosomal and mitochondrial DNA) and microbial DNA.

### Sepsi*PURE* bacterial enrichment

To establish the limit of detection, spiked 5mL blood samples were subjected to Seps*iPURE* microbial isolation and enrichment protocol (Momentum Bioscience Ltd) by simulating a 1-h transportation phase (mimicking time between phlebotomy and the beginning of sample processing) in 5mL TRM media (20˚C, static) and then combining the blood with 715μL of binding buffer containing universal microbial capture magnetic beads and incubated for 30 min for microbial extraction. Magnetic beads with microbes attached were extracted and the retained microorganisms were finally subjected to a 4-h growth phase at 37˚C in 150μL LB. Beads were removed and the media containing the enriched bacteria was mixed with PBS for a total volume of 1mL and subjected to saponin-based host DNA depletion. Then, samples underwent the scaled-up saponin-based protocol described previously (Saponin host-depletion protocol optimisation – ‘scaled-up protocol’). To individually test the Sepsi*PURE* and saponin-based methods and the effects of combining them, 5mL blood samples were spiked with 5–10 CFU/mL of *E. coli* and assessed by qPCR by measuring the effects of these methods on both host DNA (chromosomal and mitochondrial DNA) and microbial DNA.

### DNA extraction protocol

Following host DNA depletion, samples were transferred to a 2.0mL Eppendorf tube and pelleted by centrifugation at 8000xg for 6 min. Supernatants were discarded and pellets were resuspended in 1mL of PBS and transferred to 1.5mL Eppendorf tubes. Samples were pelleted again at 12,000xg for 3 min. Pellets were finally resuspended in 600μL PBS and transferred into Lysing Matrix E 2.0mL tubes (MP Biomedicals) and bead-beaten at 6m/s for 60 s on a FastPrep-24 5G instrument (MP Biomedicals) to lyse bacterial cells. After bead beating, samples were centrifuged at 20,000xg for 1 min and the supernatants containing DNA transferred into 1.5mL Eppendorfs containing 20μL of Proteinase K (Qiagen). Finally, the mixture was incubated at 65˚C for 5 min at 1000rpm. The DNA was extracted with the Maxwell RSC PureFood Pathogen kit (Promega) using the Maxwell RSC 48 (Promega) machine and finally eluted in 50μL of Elution Buffer, following the manufacturer’s protocol with the following modification: samples were directly mixed with 300μL of Lysis buffer and then transferred into the cartridge. DNA extraction protocol efficiency was assessed by first running qPCR on pure gDNA yields from *E. coli* and *S. aureus* equivalent to 10^6^ – 1 cells. The following formula was used to determine the amount of DNA to add for the cell equivalences: 1 cell genome mass = dsDNA length x bp Dalton weight. Average bp was estimated to be 615.9 Daltons and a single Dalton 1.66 × 10^−12^pg [39]. To estimate the weight of single cells, *E. coli* and *S. aureus* were considered to have genome sizes of 5Mb and 2.8Mb respectively. Running these titrated equivalences on qPCR also resulted in the generation of a regression line. Then, blood samples were spiked with 10^1^, 10^2^ and 10^5^ CFU of both *E. coli* and *S. aureus*, and saponin-based depletion and DNA extraction protocols were used. Extractions were run on qPCR and Ct (Cycle threshold) results were compared to the ones obtained from the gDNA regression line samples to check for possible loss of bacterial DNA during the extraction protocol.

### qPCR assay

SYBRGreen and probe-based qPCR assays were performed on samples which targeted human chromosomal DNA (*RNA Pol. A* gene), human mitochondrial DNA (*MT-TL1* gene), *E. coli* DNA (*cyaA* gene) and *S. aureus* (*eap* gene) (Additional File: Table S1). For all qPCR assays, each reaction contained a final concentration of 0.5μM of both reverse and forward primer, 0.2μM of probe primer (in probe-based assays for human chromosomal and *E. coli* DNA), 10μL of LightCycler 480 Probe or SYBR Green I Master Mix (2X, Roche), 5μL of DNA template and nuclease-free water was added to make a final reaction volume of 20μL. Where appropriate, qPCR Ct results were recalculated to obtain the approximate Ct values of the original samples. This was done by assuming a 100% amplification efficiency in all assays and subtracting 3.3 Cts to the obtained results. qPCR assays were performed using the LightCycler 480 instrument (Roche). Program conditions were: pre-incubation at 95°C for 5min, amplification for 50 cycles at 95°C for 30s, 55°C for 30s and 72°C for 30s, with a final extension at 72°C for 5min. Ct values were determined using the Roche LightCycler 480 software. Host DNA depletion and bacterial enrichment efficiency were represented and measured as fold change ratio values. These were calculated from the ΔCt by normalizing all values to one of the untreated control biological replicate Ct. Normalized Ct values were converted to fold change from the Eq. 2^−ΔCt^. From these data were obtained the p-value with a 2-tail unpaired t-test among different replicate groups.

### Whole genome amplification (WGA) and DNA debranching

Depleted DNA samples were subjected to WGA to increase bacterial DNA yield. Before WGA, samples were concentrated to a final eluted volume of 15μL by mixing with 1.8X of AMPure Beads (Beckman Coulter). Samples with beads were mixed using a HulaMixer (Life Technologies) and beads pelleted using a DynaMag-2 magnetic rack (Thermo Fisher). Two 200μL 70% ethanol washes were applied. Finally, DNA was eluted by resuspending the beads in nuclease-free water (Thermo Fisher). Purified and concentrated samples were amplified using the Repli-g Single Cell kit (Qiagen) according to instructions with the following adjustments: 15μL of sample was mixed with 0.16μL of 1M DTT and 1.84μL DLB Buffer at a final volume of 2μL per sample; polymerase master mix was prepared by mixing 29μL of Single Cell reaction buffer with 2μL of polymerase; amplification occurred at 37˚C for 1 h and 30 min. This optimised version of the protocol was compared to the standard protocol following the manufacturer’s handbook on purified genomic DNA. To assess the WGA efficiency of the optimised protocol and compare it to the standard version, different concentrations of extracted gDNA of both *E. coli* and *S. aureus* were added as template for the WGA reaction equivalent to adding 10^2^, 10 and 1 cells. The amplification incubation time was 2 h 30 min and samples were taken at 0, 1, 1.5, 2 and 2.5 h and ran on qPCR for the detection of *E. coli* and *S. aureus* DNA. Ct results were converted to their cell equivalences of genomic material produced by using the following equation: $Cell quantity equivalence={10}^{\frac{Ct-b}{m}}$, *m* and *b* values were taken from the regression line equation obtained in the DNA extraction experiments detailed previously. The number of *E. coli* reads obtained from blood samples spiked with 50–100 CFU of *E. coli* and subjected to saponin-based depletion, DNA extraction and WGA were compared to non-WGA controls. WGA qPCR and sequencing results were further analysed to obtain the p-value in a 2-tail unpaired t test among the different replicate groups. After WGA, artificial constructs that could interfere with nanopore sequencing were debranched using 20U of T7 Endonuclease I (NEB) and mixed with Buffer 2.0 (NEB) at 1X. Samples were incubating at 37˚C for 30 min. After debranching, samples were cleaned with AMPure Beads at 0.8X ratio and the rest of the protocol performed as per described for cleaning samples pre-WGA.

### Library preparation and MinION sequencing

Library preparation of WGA samples was completed using the Rapid PCR barcoding Kit SQK-RPB004 (Oxford Nanopore Technologies) for *‘1mL standard protocol’* samples due to typically low DNA yields following host depletion, and for samples processed through the *5mL quick-enrichment’* protocol, the Rapid Barcoding Kit SQK-RBK004 (Oxford Nanopore Technologies) was utilised because of the increase in DNA yields arising from the enrichment phase. Rapid PCR Barcoding kit library preparations followed the manufacturer’s instructions with the following modifications: up to 10ng DNA was used as template in a maximum total volume of 7.5μL with water, then 2.5μL FRM was added; reaction volumes were doubled, using 2μL RLB barcode, 50μL LongAmp Taq 2 × Master mix (NEB) and 38μL nuclease-free water (100μL total reaction volume); thermocycling parameters were initial denaturation 3 min at 95°C (1 cycle), 25 cycles of denaturation 15 s at 95°C, annealing 15 s at 56°C, extension 4 min at 65°C, then a final extension 4 min at 65°C (1 cycle) held at 4°C. The RBK protocol followed the manufacturer’s instructions except that 600ng of template DNA were added per barcode. Once libraries were prepared, samples were quantified, pooled in equal concentrations, and cleaned with a 0.6X AMPure XP bead wash eluting 10μL of 10mM Tris–HCl pH7.5–8.0 with 50mM NaCl. Approximately 100fmoles of pooled PCR library was loaded onto the MinION flow cell (R9.4.1, Oxoford Nanopore Technologies) and the entirety of the pooled RBK library were loaded according to manufacturer’s protocol. The sequencing run time was 3 h for the taxonomic identification experiments and 3 and 24 h for the AMR experiments and fastq files to be obtained.

### Quality Control—DNA quantification and fragment size measurement

To determine the quantities to add into library preparation protocols, DNA was quantified on the Qubit 4.0 (Thermo Fisher) using the high-sensitivity dsDNA kit (Thermo Fisher). The DNA libraries quality and fragment size measurement was assessed using the Genomic ScreenTape (Agilent Technologies) on the TapeStation 2200 (Agilent Technologies). DNA concentrations and fragment size measurement were also performed as such for NTC samples.

### Bioinformatics analysis

A custom pipeline for taxonomic profiling and screening antimicrobial resistance determinants was developed (https://github.com/quadram-institute-bioscience/nf-ont-taxamap). The fast5 files acquired were base-called with the Super Accuracy (SUP) model using guppy_basecaller and subsequently demultiplexed using guppy_barcoder of the Guppy software v6.2.x [40]. For the SQK-RBK004 kit, the minimum barcoding score was set at 65 and mid-read barcode filtering enabled, while for the SQK-PBK004, the barcoding score was kept at 60, and set enabled for the options: barcode both ends and mid-read barcode filtering. The pipeline involved the following steps for taxonomic profiling and screening AMR genes: human reads were removed from each barcode fastq file by mapping each barcode's reads against the *Homo sapiens* reference genome CHM13v2 [41] using minimap2 (version 2.17) [42]. Un-mapped reads to the CHM13v12 were included for mapping against the RefSeq database curated as of the 6th of March 2022 using minimap2 (v2.17). The mapped reads were filtered based on a taxa_score defined as a harmonic mean of identity and coverage of the mapped reads (2*[identity + coverage]/[identity*coverage]), only reads with taxa_score ≥ 85 (in a range from 0 to 100) were included for taxonomic profiling, which identified the taxa and their relative abundance. The non-human reads were also used for screening AMR genes using KMA (version 1.4.9) [43] tool with the chosen database amrfinderplus (v3.10). Average read coverage was calculated taking into account both upstream and downstream flanking regions and barcodes introduced during nanopore library preparation. These, combined, added approximately 100-150bp on each read which would represent approximately between 6 and 12% of the bases on each read (averaging between 1000 and 2000 bases) so maximum average read coverage values matching the reference that could be obtained were approximately 88–95%. Final results were aggregated into an Excel format, including taxa relative abundance, AMR genes, and species-based coverage estimated from the total mapped bases against multiple references of the species and then divided by the species genome size. From the relative abundance of the bacteria within each barcode/sample, the sample is concluded to be positive with the taxon if the abundance of reads for the species, over the total population of classified reads, was ≥ 90% and ≥ 5 reads matched that species. AMR genes were considered to be present if their template identity and coverage and their depth of coverage reported by KMA were superior to ≥ 98% and ≥ 1X, respectively.

## Results

### Increasing saponin concentration and sample volume, and adding bacterial enrichment improves the efficiency of host depletion

**Fig. 2:**
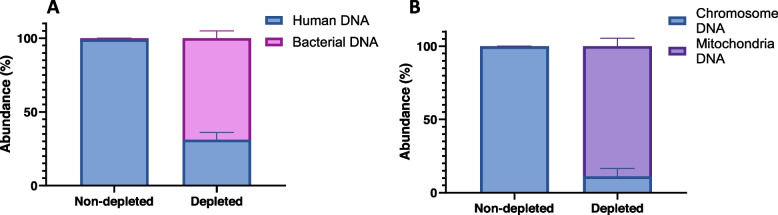
Relative abundance of sequences after saponin-based host depletion of blood samples. Samples were spiked with 50–100 CFU/mL E. coli and subjected to standard saponin-based depletion method, followed by DNA extraction and nanopore sequencing. All sequenced reads were taxonomically classified. **A** Relative abundance (%) of human chromosomal or bacterial (any species) DNA in samples after host depletion compared to control samples not subjected to host depletion (**B**) Relative abundance of human chromosomal and mitochondrial DNA after host depletion compared to control samples not subjected to host depletion. Data are means ± SD. *n* = 3 are biological replicates

A host DNA depletion step is essential to detect bacterial DNA in blood samples using metagenomic sequencing. Using whole blood samples spiked with 50–100 CFU of *E. coli*, saponin-based host depletion reduced host DNA from 99.1% of the total population of reads in non-depleted samples to 31.1% in host-depleted samples, while the proportion of bacteria reads increased from 0.86% to 68.8% (Fig. 2A). Despite the large proportional increase of bacteria DNA reads after host depletion, host DNA is still a significant component of the processed samples. The composition of the remaining host DNA was analysed and categorised as chromosomal or human mitochondrial DNA. Comparing samples before and after saponin-based depletion, 88.8% of the total population of reads matched the human mitochondrion in depleted samples, whereas in non-depleted samples human mitochondrial DNA only represented 0.11% of the classified reads (Fig. 2B).

**Fig. 3:**
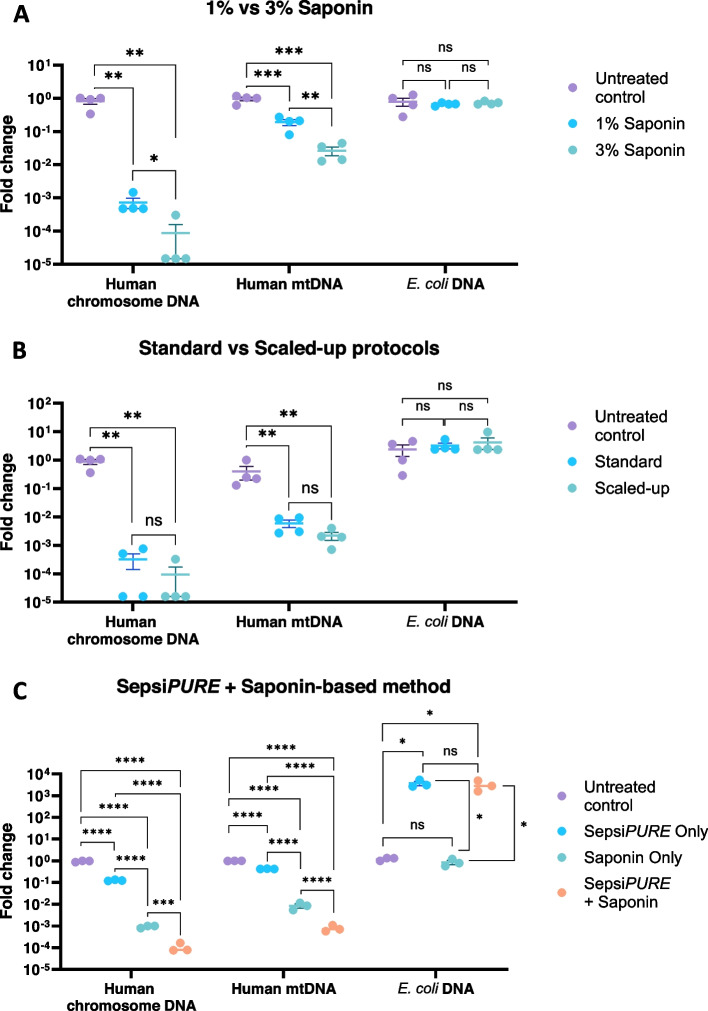
Host DNA depletion efficiency during methodological developments of saponin concentration, reaction volume and Sepsi*PURE* addition. **A** Relative quantification of human chromosomal, human mitochondrial and *E. coli* DNA in blood samples spiked with 50–100 CFU/mL *E. coli* and subjected to standard (1% saponin final concentration) and increased saponin (3%) depletion protocols. **B** Relative quantification of human chromosomal, human mitochondrial and *E. coli* DNA blood samples spiked with 50–100 CFU/mL *E. coli* and subjected to saponin-based depletion following either the Standard (200µl blood sample) or the Scaled-up (1mL blood sample) protocol. **C** Relative quantification of human chromosomal, human mitochondrial and *E. coli* DNA in blood samples spiked with 5–10 CFU/mL *E. coli* that were subjected to either Sepsi*PURE* protocol only, saponin-based method only and Sepsi*PURE* or saponin-based depletion methods combined. All values were normalised by obtaining their fold change, which represents the difference to an untreated control as measured by qPCR. Data are means, ± SD. A and B *n* = 4, C *n* = 3 are biological replicates. ns > 0.05, *p ≤ 0.05, **p ≤ 0.01. ***p ≤ 0.001, ****p ≤ 0.0001

With the efficiency of host depletion impacted by a resilient mitochondrial DNA fraction, saponin concentration was increased from 1 to 3% to lyse mitochondrial membranes more efficiently. Depletion efficiency was quantified using qPCR and represented as fold change from untreated to treated samples. Comparing the standard use of 1% saponin to 3% saponin, the depletion of each of chromosomal and mitochondria host DNA improved a further tenfold with 3% saponin (Fig. 3A). Increasing saponin concentration did not result in loss of spiked *E. coli*.

The method was further optimised by scaling up the sample volume from 200μL to 1mL to obtain a higher input of microbial cells without impacting host depletion. Using qPCR, the fold change in both host depletion or recovered *E. coli* DNA between the standard and scaled-up protocols were not significantly different (Fig. 3B). To further increase the method’s sensitivity and potentially the host depletion efficiency, the saponin-based method was combined with a bacterial enrichment method (Sepsi*PURE*). The addition of the Sepsi*PURE* enrichment step increased detection of bacterial DNA by ~ 10^3^ fold and increased the depletion of host DNA by ~ tenfold compared to unenriched samples (Fig. 3C). Alternatively, with Sepsi*PURE* alone (no saponin) there was still a ~ 10^3^-fold increase in bacterial DNA, but a marginal level of host DNA depletion compared to non-depleted samples.

### The optimised DNA extraction protocol for blood samples caused no loss of bacterial DNA when bacterial species were at clinically relevant concentrations

The optimized DNA extraction protocol was evaluated with qPCR in blood samples that had been spiked with 10^1^, 10^2^ and 10^5^ CFU of both *E. coli* and *S. aureus* from pure culture of both species (Additional File 1: Fig. S1)*.* Extracted samples were compared to bacterial gDNA yields (equivalent to 10^6^ – 1 cells) from both species. No bacterial DNA was lost in samples where low concentrations of bacteria (1 CFU) were spiked and then subjected to the saponin-based depletion and DNA extraction protocol.

### Optimised whole genome amplification significantly increases the presence of microorganism gDNA to perform library preparation

**Fig. 4:**
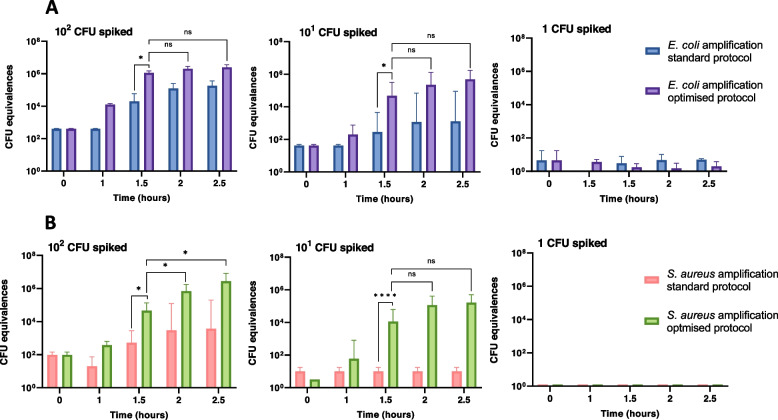
Amplification efficiency comparison for standard and optimised WGA protocols with *E. coli* and *S. aureus*. Different gDNA concentrations equivalent to adding 10^2^–1 CFU of *E. coli* (**A**) or *S. aureus* (**B**) were subjected to the standard (handbook protocol) and optimised WGA protocols. To compare both methods, samples taken at different time points were ran on qPCR and Ct values were converted to their CFU equivalences. Data are means ± SD. A and B n = 3 are biological replicates. ns > 0.05, *p ≤ 0.05, **p ≤ 0.01. ***p ≤ 0.001, ****p ≤ 0.0001

To support the application in diagnostics, the WGA step was modified by increasing the input volume of sample and also addition of DTT to the DLB Buffer. The aim was to generate a protocol that would amplify the lowest possible amount of starting bacterial DNA in the shortest possible time. Sensitivity and efficiency of the modified WGA protocol was tested and compared to the ‘standard protocol’ (following manufacturer’s instructions) and using gDNA templates of *E. coli* and *S. aureus* equivalent to adding, 100, 10 and 1 CFU. The WGA protocol was first attempted in the absence of blood and evaluated by qPCR and then converted to CFU equivalences. The optimised WGA method produced high quantities of DNA within 1.5 h of incubation when DNA was added at approximately 10 cells equivalence (Fig. 4A and 4B). When the amount of gDNA template added was equivalent to 10^2^ CFU and 10^1^ CFU of *E. coli*, the optimised WGA protocol amplified DNA to approximately 10^4^-fold increase compared to the initial input. Alternatively, results obtained with the standard protocol were significantly lower: for samples spiked with 100 and 10 CFU the DNA were only amplified a further 10^2^ and 10^1^-fold, respectively, and the amount of amplified DNA reached its peak at the 1.5 h timepoint; longer incubation times did not significantly increase the quantity of bacterial yield. Finally, amplification could not be detected in samples treated with either optimised or standard protocol when 1 CFU equivalent gDNA was used (Fig. 4A). For *S. aureus,* initial template amounts equivalent to 10^2^ CFU and 10 CFU were amplified approximately 10^3^ and 10^2^-fold, respectively, with the optimised protocol (Fig. 4B). Alternatively, amplification results obtained with the standard protocol were significantly lower with only the 100 CFU input samples DNA being amplified approximately 10X and no amplification at 10 and 1 CFU input added samples. Similar to *E. coli*—the amount of DNA amplified with the WGA optimised protocol reached its peak at the 1.5 h for *S. aureus*.

The optimised WGA protocol was assessed in the context of blood by incorporating 50–100 CFU of *E. coli* into whole blood samples. After processing mock samples using 3% saponin, libraries were prepared and sequenced for 3 h after including or omitting WGA. Inclusion of the WGA protocol significantly increased the number of bacterial reads from extracted blood samples compared to the same samples with no WGA step. In the absence of WGA, an average of 4.8 ± 3.8 reads were classified as *E. coli*. Subjecting the same samples to a WGA step resulted in an average of 7126.3 ± 2460.0 reads taxonomically classified as *E. coli*, which constitutes a significant > 10^3^-fold increase in *E. coli* reads.

### *E. coli*, S*. aureus*, *K. pneumoniae* and *E. faecalis* can be detected at clinically relevant concentrations

Different concentrations of *E. coli*, S*. aureus*, *K. pneumoniae* and *E. faecalis* were spiked into 1mL or 5mL of blood and samples were subjected to *‘1mL standard protocol’* and ‘*5mL quick-enrichment protocol’* and then analysed with a custom bioinformatics pipeline (see Fig. 1).

**Fig. 5:**
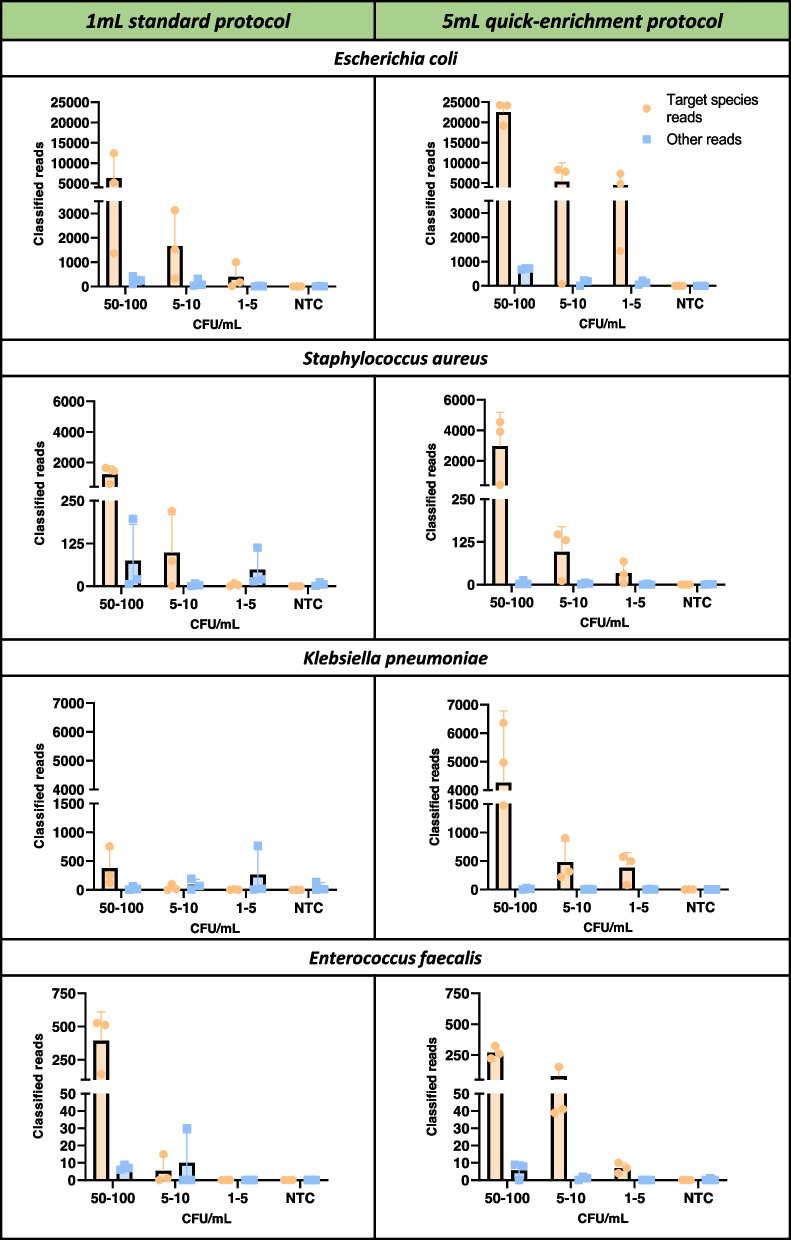
Bacterial sequence reads with the 1mL and 5mL CMg pipelines*.* Number of classified reads obtained on *E. coli, S. aureus, K. pneumoniae and E. faecalis* when spiked at different concentrations (50–100, 5–10 or 1–5 CFU/mL). All species and concentrations were spiked into 1mL or 5mL of whole blood and were subjected to whole standard and quick-enrichment pipelines. Sequencing results were grouped into two different categories: *Target species reads* = taxonomically classified reads that matched the spiked species; *Other reads* = reads that were classified as any other microbial species. Data are means ± SD. *n* = 3 are biological replicates

Within the analysis pipeline, a threshold for the minimum relative abundance of reads and minimum number of reads and was established by reviewing the distribution of reads contributed from spiked species versus those attributed to likely contaminating species (see Additional files 1: Tables S2 and S3). After the application of the taxonomic thresholds (≥ 90% abundance, ≥ 5 total number of identified reads), the limit of detection of the *‘1mL standard protocol’* was 1–5 CFU/mL for *E. coli*, 5–10 CFU/mL for *S. aureus,* and 50–100 CFU/mL for *K. pneumoniae* and *E. faecalis* (Fig. 5; Table 1). The concentration of spiked bacteria correlated with the total number of obtained sequenced reads, which varied amongst the input species (from an average of 5 to more than 6K reads). Given that only known bacterial species were spiked into blood samples yet these reference species accounted a minority of bacterial reads in some samples (e.g., the 1–5 CFU/mL *K. pneumoniae and* 1–5 CFU/mL *E. faecalis* samples), the taxonomic assignment of the remaining bacterial reads was further examined (Additional file 1: Table S2). Reads representing possible contaminants contributed by skin flora or reagent contaminants were detected (e.g., *Cutibacterium acnes*) but no contaminants represented ≥ 90% of the bacterial population relative abundance. Further, none of the NTC samples were positive for any taxonomically classified microorganism species after applying the thresholds (Additional file 1: Table S3). This entire pipeline took approximately 9 h from sample collection to results.

**Table 1:** Sequencing metrics for the 1mL and 5mL CMg pipelines

	**Total number of reads matching reference**		**Bacteria population relative abundance (%)**		**Average bp length of mapped reads to reference (average read coverage(%))**		**Average coverage to reference (X)**	
	**1 mL protocol**	**5 mL protocol**	**1 mL protocol**	**5 mL protocol**	**1 mL protocol**	**5 mL** **protocol**	**1 mL protocol**	**5 mL protocol**
***Escherichia coli***								
** 50–100 CFU/mL**	6306.7 ± 3238.1	22,516.3 ± 1677.5	95.2 ± 0.9	96.9 ± 0.2	1878.8.3 ± 129.4 (91.0 ± 0.6)	1132.3 ± 54.5 (92.1 ± 4.0)	2.4 ± 1.25	4.7 ± 0.5
** 5–10 CFU/mL**	1662.3 ± 811.9	5384.7 ± 2655.6	90.5 ± 4.4	95.9 ± 1.4	2221.4 ± 108.9 (92.2 ± 0.4)	1197.1 ± 26.6 (88.2 ± 0.2)	0.7 ± 0.4	1.1 ± 0.6
** 1–5 CFU/mL**	392.7 ± 306.8	4521.3 ± 1701.9	94.9 ± 1.2	96.9 ± 0.2	2519.2 ± 71.8 (89.1 ± 3.6)	1148.9 ± 12.2 (87.8 ± 0.1)	0.2 ± 0.1	0.9 ± 0.3
***Staphylococcus aureus***								
** 50–100 CFU/mL**	1224.7 ± 320.6	2963.0 ± 1279.6	98.9 ± 3.6	99.7 ± 0.1	1947.7 ± 117.3 (90.6 ± 0.4)	1336.5 ± 12.6 (89.0 ± 0.2)	0.8 ± 0.21	0.9 ± 0.4
** 5–10 CFU/mL**	98.0 ± 64.4	96.0 ± 42.8	96.2 ± 3.1	95.1 ± 1.8	2496.2 ± 390.1 (85.7 ± 6.0)	687.2 ± 21.2 (93.6 ± 2.3)	0.06 ± 0.04	0.02 ± 0.01
** 1–5 CFU/mL**	4.0 ± 2.6	34.0 ± 18.6	17.4 ± 3.2	98.6 ± 1.4	1892.7 ± 17.9 (91.6 ± 0.7)	797.1 ± 83.8 (94.7 ± 1.7)	0.002 ± 0.001	0.005 ± 0.002
***Klebsiella pneumoniae***								
** 50–100 CFU/mL**	377.7 ± 192.4	4268.7 ± 1452.8	94.6 ± 1.6	99.3 ± 0.6	2481.8 ± 587.2 (92.0 ± 1.3)	1379.9 ± 55.6 (89.5 ± 0.4)	0.06 ± 0.02	2.7 ± 1.5
** 5–10 CFU/mL**	39.3 ± 31.1	479.3 ± 213.7	21.4 ± 6.7	98.9 ± 1.1	2219.5 ± 263.7 (92.5 ± 1.6)	1262.6 ± 50.3 (88.7 ± 0.2)	0.003 ± 0.002	0.1 ± 0.05
** 1–5 CFU/mL**	4.7 ± 3.3	382.3 ± 152.7	5.5 ± 4.1	95.3 ± 4.6	2345.9 ± 31.8 (93.9 ± 0.1)	1216.5 ± 49.6 (88.5 ± 0.4)	0.0007 ± 0.0	0.07 ± 0.03
***Enterococcus faecalis***								
** 50–100 CFU/mL**	393.7 ± 125.4	268.0 ± 29.3	97.2 ± 0.8	97.9 ± 1.0	2350.3 ± 138.9 (91.3 ± 0.6)	1161.3 ± 66.7 (92.1 ± 4.0)	0.26 ± 0.07	0.04 ± 0.02
** 5–10 CFU/mL**	5.4 ± 4.8	78.0 ± 38.0	44.5 ± 29.4	98.2 ± 1.4	2474.4 ± 181.7 (90.6 ± 0.6)	1091.0 ± 109.9 (97.6 ± 0.3)	0.006 ± 0.005	0.008 ± 0.004
** 1–5 CFU/mL**	0.0 ± 0	7 ± 1.7	0.0 ± 0	100 ± 0	-	1107.7 ± 377.4 (96.3 ± 1.1)	-	0.002 ± 0.001

Sequencing results obtained in experiments on 1mL and 5mL of blood spiked with 50–100, 10–50 and 1–5 CFU/mL of *E. coli, S. aureus, K. pneumoniae and E. faecalis*. *Total number of reads matching reference* = number of reads matching species reference species spiked. *Bacteria population relative abundance (%)* = abundance of targeted species reads over the total number of taxonomically classified reads obtained. *Average bp length of mapped reads to reference (average read coverage (%))* = average length of the reads that matched the reference and percentage of identical nucleotides per read between template and consensus (spiked species). *Average coverage to reference (X)* = estimated depth of coverage taking into account the number of reads matching the reference and their average length. Data are means ± SD. *n* = 3 are biological replicates

When pathogens were spiked at different concentrations in 5mL blood samples and tested with the ‘*5mL quick-enrichment protocol’*, pathogens were detected at all concentrations, equating to a sensitivity of 1–5 CFU/mL across the four species (Fig. 5)***.*** For all test species and at all concentrations the average proportion of bacterial reads matching their appropriate reference was ≥ 90%, and the number of sequence reads matching the reference were ≥ 5, which indicates a limit of detection of 1–5 CFU/mL for this pipeline. Similar to the 1mL standard protocol, the spiked inoculum of the four species directly correlated with the total number of obtained reads, although this varied among species (from an average of 5 to over 20K matching their respective references). In some samples, the number of classified reads was minimal (between 5–100 reads; Fig. 5; Table 1). Despite this, their relative proportion of classified reads was consistently high (~ 95–100%), outnumbering the number of reads from any other species. With the introduction of the Sepsi*PURE* step, this pipeline required approximately 12 h to completed from sample collection to results. Consistent with the taxonomic results obtained from 1mL samples, all the spiked species were completely absent in their respective NTCs (Additional file 1: Table S3).

We further analysed the mapped reads to obtain data on read and genomic coverage (Table 1). Amplicon sizes averaged 1.5–2.5Kbp and 1–1.5Kbp for the *‘1mL standard protocol’* and *‘5mL quick-enrichment’* pipelines, respectively, and averaged 88%-95% matching the reference for both pipelines, which matches the theoretical maximum since sequences included barcode sequences introduced during library preparation. With both protocols, the average genome coverage was directly proportional to the concentration of spiked bacteria (Table 1). For the *‘1mL standard protocol’*, a lower number of reads resulted on low coverage values ranging from an average of approximately 1X coverage at 50–100 CFU/mL to 0.001X coverage at 1–5 CFU/mL. The ‘*5mL quick-enrichment protocol’* produced approximately 10X more reads than the *‘1mL standard protocol’*, however, this did not result in a proportionate increase in coverage when comparing the protocols. Across all species, average coverage ranged from approximately 5X at highest concentrations spiked to 0.005X at the lowest.

### Antimicrobial resistance determinants could be identified at all spiked concentrations for *E. coli*.

Different concentrations of β-lactamase resistant *E. coli* CXTM-15 (containing the pEK499 plasmid which encodes 10 AMR determinants) were spiked into 1mL or 5mL of blood to determine if AMR determinants are detectable using any of the two presented CMg pipelines. When using the *'1mL standard protocol’* pipeline, 6 of 10 AMR determinants could be detected on samples spiked at the highest concentration (100 CFU/mL), 2 at the 10 CFU/mL concentration, and none at the lowest spiked concentrations. When detected, average coverage was low (< 10X) but the high template identity (≥ 98%,) and high template coverage values (between 100–102%) confirmed the presence of all these AMR genes (Table 2). When assessing the *‘5mL quick-enrichment protocol' samples,* all resistance genes could be detected at any input concentration, except for *tet(A)*, *catB4* and *blaTEM-1* at the lowest concentrations. On two occasions, genes were misidentified: *aac(6’)-lb-W104R, aac(6’)-lb-D181Y* and *aac(6’)-lb-AKT* instead of the expected *aac6’-lb-cr;* and *catB3* instead of the expected *catB4*. Average coverage obtained in samples spiked with low amounts of bacteria (1–10 CFU/mL) was generally low (< 10X). However, the high average template identity (≥ 99%) and coverage (between 99–101%) values confirmed the presence of all these genes when using the *‘5mL quick-enrichment protocol'*.

**Table 2:** Sequencing metrics of AMR genes using the 1mL and 5mL CMg pipelines

pEK499 genes	Average Coverage (X)						Average template identity (%) / Average Template Coverage (%)					
	**1 mL protocol**			**5 mL protocol**			**1 mL protocol**			**5 mL protocol**		
	**50–100 CFU/mL**	**5–10 CFU/mL**	**1–5 CFU/mL**	**50–100 CFU/mL**	**5–10** **CFU/mL**	**1–5** **CFU/mL**	**50–100 CFU/mL**	**5–10** **CFU/mL**	**1–5** **CFU/mL**	**50–100 CFU/mL**	**5–10 CFU/mL**	**1–5 CFU/mL**
***blaCTX-M-15***	4.1 ± 0.7	1.0 ± 1.0	0	48.9 ± 10.1	11.3 ± 1.8	17.3 ± 7.5	100.0 ± 0 / 100.0 ± 0	100.0 ± 0 / 100.3 ± 0	-	99.0 ± 0.0 / 100.0 ± 0	100.0 ± 0 / 100.0 ± 0	99.0 ± 0.1 / 100.8 ± 0.8
***blaOXA-1***	0	0	0	6.4 ± 1.4	2.0 ± 1.0	5.5 ± 2.8	-	-	-	100.0 ± 0 / 100.0 ± 0	99.9 ± 0.1 / 100.0 ± 0	100.0 ± 0 / 100.8 ± 0.8
***blaTEM-1***	1.0 ± 0.1	0	0	2.7 ± 0.1	0.7 ± 0.7	0	98.8 ± 0.3 / 100.5 ± 0.1	-	-	99.7 ± 0 / 101.7 ± 1.2	99.4 ± 0.0 / 101.4 ± 1.4	-
***aac6’-lb-cr***	0	0	0	3.9 ± 2.9	1.7 ± 0.9	1.9 ± 1.1	-	-	-	99.8 ± 0.0 / 100.3 ± 0.3	98.9 ± 1.1 / 100.3 ± 0.3	99.7 ± 0.1 / 100.5 ± 0.5
***mph(A)***	5.6 ± 2.6	1.0 ± 0.6	0	39.8 ± 6.5	6.9 ± 1.5	13.0 ± 7.7	99.4 ± 0.6 / 100.0 ± 0	100.0 ± 0 / 100.3 ± 0	-	100.0 ± 0 / 100.0 ± 0	100.0 ± 0 / 100.0 ± 0	100.0 ± 0 / 100.0 ± 0
***catB4***	0		0	7.3 ± 1.7	1.4 ± 0.2	0.7 ± 0.7	-	-	-	92.5 ± 1.2 / 102.1 ± 0.7	86.9 ± 1.0 / 101.7 ± 1.0	94.5 ± 0 / 99.8 ± 0
***tet(A)***	2.3 ± 2.3	0	0	0.3 ± 0.3	0	0.3 ± 0.3	92.5 ± 0 / 101.7 ± 0	-	-	98.3 ± 0.0 / 100.2 ± 0	-	73.2 ± 0 / 77.4 ± 0
***dfrA7***	4.5 ± 1.5	0	0	16.5 ± 2.6	5.7 ± 0.3	3.5 ± 1.9	99.1 ± 0.8 / 100.1 ± 0.1	-	-	99.8 ± 0.0 / 100.0 ± 0	99.8 ± 0.0 / 100.0 ± 0	99.3 ± 0.5 / 100.1 ± 0.1
***sulI***	3.6 ± 2.0	0	0	18.7 ± 5.8	3.6 ± 1.6	3.6 ± 2.0	99.8 ± 0.2 / 100.1 ± 0.1	-	-	100.0 ± 0 / 100.1 ± 0	98.9 ± 1.2 / 100.0 ± 0	99.9 ± 0.1 / 100.1 ± 0.1
***aadA5***	4.9 ± 0.1	0	0	19.6 ± 3.8	9.1 ± 0.5	4.4 ± 2.6	99.0 ± 0.1 / 100.0 ± 0	-	-	99.9 ± 0.1 / 100.0 ± 0	100. ± 0.0 / 100.0 ± 0	100. ± 0 / 100.0 ± 0

1mL or 5mL of blood were spiked with *E. coli* CTX-M-15 (peK499 plasmid) at different concentrations (50–100, 10–50 and 1–5 CFU/mL) and subjected to ‘*1mL standard protocol’* and *‘5mL quick-enrichment protocol’*. AMR determinants were detected and samples were compared by assessing their coverage and template identity to the reference. *Average Coverage (X)* = average depth of coverage of the template. *Average template identity (%)* = percentage of identical nucleotides between template and consensus. *Average template coverage (%)* = percentage of bases in the template that is covered by consensus sequence. Data are means ± SD. *n* = 3 are biological replicates

The potential benefits of extending the sequencing time for AMR determinant detection were examined. We introduced varying concentrations of the *E. coli* CXTM-15 strain into 5mL blood samples, followed the*’5 mL quick-enrichment protocol’,* and subjected them to 24-h sequencing. These outcomes were compared against samples sequenced for 3 h (as shown in Table 2). The longer sequencing times noticeably increased gene coverage across all targets (Additional file 1: Table S4). This enabled successful detection of the *blaTEM-1* gene across all spiked concentrations, which remained elusive during the initial 3-h sequencing. Conversely, as per the initial 3-h sequencing results, the *catB3* and *tet(A)* genes remained undetected. The extended sequencing time led to reduce the misidentification of genes from the pEK499 plasmid. Noticeably, only *aac(6’)-lb-*W104R was identified in one replicate instead of the expected *aac6’-lb-cr*. Similarly, *catB4* was consistently detected across all replicates, as opposed to the expected *catB3* gene.

## Discussion

Culture-based approaches have been the long-standing gold standard method for BSI diagnostics. Although there have been some enhancements in terms of sample throughput, improvements to the overall sensitivity and time to detection have been limited [44]. CMg is a promising new approach for many microbiological diagnostics, however attempts to develop a sequencing-based methodology applicable to BSI have proved challenging for numerous reasons: (1) the low abundance of microorganisms compared to host cells in blood, (2) difficulties in extraction and purification of microbial DNA at this abundance (3) false positive results contributed by contamination and background DNA, (4) challenges in interpretation of sequence data for the accurate assignment of etiologic agents amongst multiple detected taxa, and (5) extended turn-around times in practice [18–22]. In this study, we developed a proof-of-concept CMg pipeline method that overcomes these issues and can detect causative species of BSI when spiked at clinically relevant levels. This method has the potential to be used in place of blood culture as a more sensitive and faster method for BSI diagnostic.

Since the publication of the use of a saponin-based host DNA depletion method in respiratory samples in 2019 [32], the method has been further improved as a streamlined version that includes a saponin final concentration of 1% [38] with the potential use for blood samples [45]. In this study, despite being highly efficient at removing host chromosomal DNA (> 99.9% removal), there was still a significant presence of human mitochondrial DNA after depleting spiked whole blood samples. The lytic effect of saponin is due to an interaction with the sterol molecules present on cell membranes [46], which are present in eukaryotic cells [47] and absent in bacteria cell membranes [48]. However, both inner and outer human mitochondria cell membranes contain a relatively low number of sterol groups [49, 50], making them more resistant to the saponin treatment. In this study, after using the standard saponin-based host depletion methodology, mitochondrial DNA remained in the sample at an abundance that impacted the detection of bacterial DNA. Thus, the resistance of mitochondria to saponin treatment decreased the sensitivity of the pipeline, highlighting the importance of improving the host mitochondrial DNA removal to increase the overall CMg pipeline sensitivity. We improved the depletion of human mitochondrial DNA by augmenting the saponin concentration as well as scaling-up the processed volume without imparting a detrimental effect on the detectable bacteria. We believe that adding a higher concentration of saponin facilitated further interaction between the detergent and the limited number of sterols on the mitochondria membranes. Depletion of mitochondria was further improved by combining the saponin-based depletion method with Sepsi*PURE* microbial capture in the *‘5mL quick-enrichment protocol’*, wherein the combination of both methods resulted in an increase in *E. coli* DNA detection as well as the highest levels of host DNA depletion, as detected by absolute DNA quantification by qPCR (Fig. 3C), indicating a its potential to yield the highest sensitivity in the subsequent metagenomics sequencing of the samples. We hypothesise that these shifts were due to selective binding of bacteria over host cells by the capture beads, which were then further enriched during a 4-h growth phase in LB broth. Alternatively, residual host genomic or mitochondrial DNA bound to beads was then removed by discarding the beads before subjecting the bacteria-containing media to saponin-based depletion. The introduction of a growth step also likely facilitated selection of intact, viable microbial cells. We acknowledge that enrichment may compromise the detection of species that are fastidious [51]**.** As such, we also present the *‘1mL standard protocol’*, a sensitive method that can directly analyse 1mL blood samples and does not require an enrichment phase.

In all versions of the protocol, after host depletion and DNA extraction, DNA was amplified using an optimised WGA protocol. Despite the potential ability of WGA to generate species-bias during amplification [52], this effect is less consequential when used on BSI samples as the majority of infections are monomicrobial [53, 54]. When gDNA from microorganisms was present at low concentration, the WGA optimised protocol could amplify large quantities of bacterial DNA from very low inputs (equivalent to approximately 10 CFU) within 1 h and 30 min. Despite the WGA protocol not resulting in demonstrable amplification for samples spiked with gDNA equivalent to 1 CFU, microbial reads were detected in blood samples spiked with 1–5 CFU/mL. We hypothesize that the molecular crowding effect during the multiple-displacement amplification [55] caused the amplification of the low number of bacterial reads which are surrounded by a high presence of host DNA.

The two variants of the methodology—one involving bacterial enrichment (*‘5mL quick-enrichment protocol’*) and one that directly analyses blood with enrichment it (*‘1mL standard protocol')—*were tested on mock blood samples spiked with four of the main causative species of BSI *E. coli*, *S. aureus*, *K. pneumoniae* and *E. faecalis* [56]*.* Final products of the WGA and debranching steps were sequenced using nanopore sequencing, which was chosen for its potential to enable rapid library preparation and provide real-time sequence results, resulting in a significant reduction in turnaround times [57]. After sequence analyses using a customised bioinformatics pipeline, results were available in 9 h and 12 h for 1mL and 5mL pipelines, respectively (Fig. 5).

Notably, the limit of detection for the methodology was 1–5 CFU/mL for *E. coli*, 5–10 CFU/mL for S*. aureus* and 50–100 CFU/mL for *K. pneumoniae* and *E. faecalis* with the *‘1mL standard protocol'* pipeline. When using the *‘5mL quick-enrichment protocol’* pipeline the analytic sensitivity was 1–5 CFU/mL for all species. The *‘1mL standard protocol’* was not sufficient to deplete host DNA to the same degree (with a significant mitochondrial DNA fraction remaining) which impacted the detection of microbial species when present at low concentrations. The different LoD values between the spiked species when using the 1mL, a potential limitation of this method, could be due to the introduction of species bias at certain steps: (1) the saponin-based method, which has been reported to potentially lyse certain bacterial species leading to a loss of their DNA [32]; (2) DNA extraction methods have differential effectiveness between bacterial species [19, 58]; (3) the WGA method or the Rapid PCR barcoding library kit, which use polymerases (phi29 or LongAmp™) that are known for having more affinity for amplifying certain bacterial species [59, 60]. If testing clinical samples (rather than spiked samples with known microbial species) it will be important to include extraction (positive) controls to confirm that productive extractions have occurred. Further, as commonly described in other metagenomic studies, this pipeline could also be susceptible to contamination and false positive results [23]. However, after the application of the appropriate analytical thresholds, all spiked samples were negative for any non-targeted species. Similarly, all the non-template control samples were negative for any bacterial species. The introduction of an enrichment phase as part of the *‘5mL quick-enrichment protocol’* resulted in increased yields of microbial DNA. Consequently, adding higher yields to the WGA reaction resulted in increased yields post-WGA when compared to non-enriched samples. This allowed the use of a rapid barcoding library instead of the PCR-based approach used in the *‘1mL standard protocol’*, which is one of the fastest and simplest manual library preparation protocols [61–63]. Nonetheless, this caused a slightly shorter average amplicon size and consequently, the augment on reads matching the reference did not result in a proportional increase in genome coverage.

Genomic coverage varied among the different spiked species and, as read length values were similar across samples, it was directly impacted by the number of reads obtained (which also varied among species). Despite having a limit of detection of 1–5 CFU/mL for all species when using the *‘5mL quick-enrichment protocol',* some species like *E. coli*, showed a significantly higher number of reads compared to other species and consequently a higher average genome coverage. We hypothesize that the short enrichment phase could be introducing species-differences due to both the quantity of bacteria bound to the beads and their multiplication during the media incubation. This, combined with all the previously listed steps of the pipeline that can potentially introduce species-bias, had a direct impact on the final number of reads and the genomic coverage and could potentially be a limitation of the protocol.

The detection of antimicrobial genes could be challenging due to the low coverage numbers obtained using the protocols (< 10X) [64], making difficult establishing phenotypic correlations [65, 66]. However, when the pipeline was spiked with different concentrations of *E. coli* CXT-M-15, the majority of resistance genes encoded by pEK499 [67] were detected. Compared to obtained genomic coverage values on LoD experiments, all resistance genes had a higher depth of coverage (≥ 1X). This, together with the high template identity and template coverage obtained, allowed the detection of almost all resistance genes at the lowest spiked concentrations. As pEK499 is a low-copy number plasmid [68, 69], its yield would not be superior to the chromosomic bacterial DNA. The increased coverage on plasmid AMR genes could be explained by the plasmid amplification bias introduced during WGA, which has also been described by other authors [59, 70]. This could be problematic when trying to detect chromosomal AMR genes. Nonetheless, the most prevalent BSI causative antimicrobial resistance strains (MRSA, Vancomycin-Resistant *Enterococcus*, Multidrug-Resistant *Enterobacteriaceae*, Extended-Spectrum β-Lactamase (ESBL) gram-negative species or Carbapenem-Resistant *Enterobacterales* [56]), generally have plasmid-mediated resistant mechanisms [71–75]. Because of the low coverage obtained in two of the genes present on the plasmid (*aac6’-lb-cr* and *catB4*), these were misidentified as closely related genes: *aac(6’)-lb-W104R, aac(6’)-lb-D181Y, aac(6’)-lb-AKT and catB3*. However, all these variants were mutations of the expected genes and are part of the same resistance mechanisms [76–78]. To overcome this, our method could be adapted to have longer sequencing incubation times, resulting in higher coverage. As nanopore sequencing allows real-time visualisation of results, we propose that within the first hours of sequencing the causative microorganisms could be identified and extended incubation times (3–36 h) would allow, thanks to the higher coverage, an improved detection of specific AMR determinants.

## Conclusion

In this study we developed a CMg pipeline for the detection of bloodborne microbial species and antimicrobial resistance genes. We demonstrated that current saponin-based host depletion methods lack the necessary depletion efficiency to detect bacterial species in blood (when these are present at low concentrations) due to a relatively high proportion of retained human mitochondria DNA. From this, we proposed that additional reduction of mitochondrial DNA would greatly improve clinical metagenomic pipelines. This was achieved with the addition of a bead-based enrichment protocol followed by a higher concentration of saponin during host depletion steps. Classification of the resulting microbial sequences was completed using a customised bioinformatics approach with a threshold for determining reportable findings, where in LoD experiments with mock samples, only true positives were observed in spiked samples and no false negatives were observed in control samples. Antimicrobial resistance genes were also detectable if there was sufficient genome coverage. As a result, and in combination with additional optimisation steps, four of the main causative species of BSI could be detected when spiked into 5mL of whole blood at clinically relevant concentrations (1–5 CFU/mL blood) in a 12-h protocol, from sample collection to results. We anticipate that further reductions to human mitochondrial DNA are possible and will have a similar or incremental effect on the sensitivity of CMg pipelines for BSI. It will also be important to test a modified version of the protocol to detect *Candida* species due to the high mortality of candidemia [79] while accounting for fungal cell wall structures containing sterols [80] which may be lysed with saponin during host depletion steps. In conclusion, we present several methodological developments and optimizations to improve a CMg pipeline that is now suited for validation with clinical patient samples in conjunction with culture and molecular diagnostic methodologies. Compared to blood culture, this metagenomic methodology could be used as a more rapid and sensitive diagnostic to improve the management of patients with BSI.

### Supplementary Information

**Supplementary Material 1. **

**Supplementary Material 2. **

## Data Availability

All the sequencing data supporting the conclusions of this article are available at the European Nucleotide Archive (ENA) repository under accession PRJJEB64522 (https://www.ebi.ac.uk/ena/browser/view/PRJEB64522). The accession and run numbers for each sample can be found on Additional file 2: Table S5.

## Abbreviations

AMR: Antimicrobial resistance
AST: Antimicrobial susceptibility testing
BC: Blood culture
BSI: Bloodstream infection
CFU: Colony forming unit
cfDNA: Cell-free DNA
CMg: Clinical metagenomics
CPD: Citrate-Phosphate-Dextrose
EDTA: Ethylenediaminetetraacetic acid
gDNA: Genomic DNA
LoD: Limit of detection
WGA: Whole genome amplification

## References

[1] Rudd KE, Johnson SC, Agesa KM, Shackelford KA, Tsoi D, Kievlan DR (2020). Global, regional, and national sepsis incidence and mortality, 1990–2017: analysis for the Global Burden of Disease Study. Lancet.

[2] Cohen J, Vincent JL, Adhikari NKJ, Machado FR, Angus DC, Calandra T (2015). Sepsis: A roadmap for future research. Lancet Infect Dis.

[3] Gupta S, Sakhuja A, Kumar G, McGrath E, Nanchal RS, Kashani KB (2016). Culture-Negative Severe Sepsis: Nationwide Trends and Outcomes. Chest.

[4] Chen P, Li S, Li W, Ren J, Sun F, Liu R (2020). Rapid diagnosis and comprehensive bacteria profiling of sepsis based on cell-free DNA. J Transl Med.

[5] Kumar A, Ellis P, Arabi Y, Roberts D, Light B, Parrillo JE (2009). Initiation of inappropriate antimicrobial therapy results in a fivefold reduction of survival in human septic shock. Chest.

[6] Kumar A, Roberts D, Wood KE, Light B, Parrillo JE, Sharma S (2006). Duration of hypotension before initiation of effective antimicrobial therapy is the critical determinant of survival in human septic shock. Crit Care Med.

[7] Bisarya R, Song X, Salle J, Liu M, Patel A, Simpson SQ (2022). Antibiotic Timing and Progression to Septic Shock Among Patients in the ED With Suspected Infection. Chest.

[8] Lamy B, Sundqvist M, Idelevich EA (2020). Bloodstream infections – Standard and progress in pathogen diagnostics. Clin Microbiol Infect.

[9] Idelevich EA, Seifert H, Sundqvist M, Scudeller L, Amit S, Balode A (2019). Microbiological diagnostics of bloodstream infections in Europe—an ESGBIES survey. Clin Microbiol Infect.

[10] Idelevich EA, Schüle I, Grünastel B, Wüllenweber J, Peters G, Becker K (2014). Acceleration of antimicrobial susceptibility testing of positive blood cultures by inoculation of Vitek 2 cards with briefly incubated solid medium cultures. J Clin Microbiol.

[11] Costa SP, Carvalho CM (2022). Burden of bacterial bloodstream infections and recent advances for diagnosis. Pathog Dis.

[12] Afshinnekoo E, Chou C, Alexander N, Schuetz AN, Mason CE (2017). Precision Metagenomics : Rapid Metagenomic Analyses for Infectious Disease Diagnostics and Public Health Surveillance. J Biomolec.

[13] Peri AM, Stewart A, Hume A, Irwin A, Harris PNA (2021). New Microbiological Techniques for the Diagnosis of Bacterial Infections and Sepsis in ICU Including Point of Care. Curr Infect Dis Rep.

[14] Hu T, Chitnis N, Monos D, Dinh A (2021). Next-generation sequencing technologies: An overview. Hum Immunol.

[15] Chiu CY, Miller SA (2019). Clinical metagenomics. Nat Rev Genet.

[16] Köser CU, Ellington MJ, Cartwright EJP, Gillespie SH, Ko CU, Brown NM (2012). Routine Use of Microbial Whole Genome Sequencing in Diagnostic and Public Health Microbiology. PLoS Pathog..

[17] Poole S, Kidd SP, Saeed K (2018). A review of novel technologies and techniques associated with identification of bloodstream infection etiologies and rapid antimicrobial genotypic and quantitative phenotypic determination. Expert Rev Mol Diagn.

[18] Forbes JD, Knox NC, Ronholm J, Pagotto F, Reimer A (2017). Metagenomics: The next culture-independent game changer. Front Microbiol.

[19] Dalla-Costa LM, Morello LG, Conte D, Pereira LA, Palmeiro JK, Ambrosio A (2017). Comparison of DNA extraction methods used to detect bacterial and yeast DNA from spiked whole blood by real-time PCR. J Microbiol Methods.

[20] Strong MJ, Xu G, Morici L, Splinter Bon-Durant S, Baddoo M, Lin Z (2014). Microbial Contamination in Next Generation Sequencing: Implications for Sequence-Based Analysis of Clinical Samples. PLoS Pathog.

[21] Gu W, Miller S, Chiu CY (2019). Clinical Metagenomic Next-Generation Sequencing for Pathogen Detection. Annu Rev Pathol Mech Dis.

[22] Schlaberg R, Chiu CY, Miller S, Procop GW, Weinstock G (2017). Validation of Metagenomic Next-Generation Sequencing Tests for Universal Pathogen Detection. Arch Pathol Lab Med.

[23] Thoendel M, Jeraldo P, Greenwood-Quaintance KE, Yao J, Chia N, Hanssen AD (2017). Impact of contaminating DNA in whole-genome amplification kits used for metagenomic shotgun sequencing for infection diagnosis. J Clin Microbiol.

[24] Sinha M, Jupe J, Mack H, Coleman TP, Lawrence SM, Fraley I (2018). Emerging Technologies for Molecular Diagnosis of Sepsis. Clin Microbiol Rev.

[25] Grumaz S, Grumaz C, Vainshtein Y, Stevens P, Glanz K, Decker SO (2019). Enhanced Performance of Next-Generation Sequencing Diagnostics Compared with Standard of Care Microbiological Diagnostics in Patients Suffering from Septic Shock. Crit Care Med.

[26] Blauwkamp TA, Thair S, Rosen MJ, Blair L, Lindner MS, Vilfan ID (2019). Analytical and clinical validation of a microbial cell-free DNA sequencing test for infectious disease. Nat Microbiol.

[27] Hong DK, Blauwkamp TA, Kertesz M, Bercovici S, Truong C, Banaei N (2018). Liquid biopsy for infectious diseases : sequencing of cell-free plasma to detect pathogen DNA in patients with invasive fungal disease. Diagnostic Microbiol Infect Dis.

[28] Dinakaran V, Rathinavel A, Pushpanathan M, Sivakumar R, Gunasekaran P, Rajendhran J (2014). Elevated levels of circulating DNA in cardiovascular disease patients: Metagenomic profiling of microbiome in the circulation. PLoS ONE.

[29] Grumaz C, Hoffmann A, Vainshtein Y, Kopp M, Grumaz S, Stevens P (2020). Rapid Next-Generation Sequencing Based Diagnostics of Bacteremia in Septic Patients. J Mol Diagnostics.

[30] O’Grady J (2019). A powerful, non-invasive test to rule out infection. Nat Microbiol.

[31] Schmidt K, Mwaigwisya S, Crossman LC, Doumith M, Munroe D, Pires C (2017). Identification of bacterial pathogens and antimicrobial resistance directly from clinical urines by nanopore-based metagenomic sequencing. J Antimicrob Chemother.

[32] Charalampous T, Kay GL, Richardson H, Aydin A, Baldan R, Jeanes C (2019). Nanopore metagenomics enables rapid clinical diagnosis of bacterial lower respiratory infection. Nat Biotechnol.

[33] Peker N, Couto N, Sinha B, Rossen JW (2018). Diagnosis of bloodstream infections from positive blood cultures and directly from blood samples : recent developments in molecular approaches. Clin Microbiol Infect.

[34] Lecuit M, Eloit M (2015). The potential of whole genome NGS for infectious disease diagnosis. Expert Rev Mol Diagn.

[35] Parize P, Pilmis B, Lanternier F, Lortholary O, Lecuit M, Muth E (2017). Untargeted next-generation sequencing-based first-line diagnosis of infection in immunocompromised adults: a multicentre, blinded, prospective study. Clin Microbiol Infect.

[36] Feehery GR, Yigit E, Oyola SO, Langhorst BW, Schmidt VT, Stewart FJ (2013). A Method for Selectively Enriching Microbial DNA from Contaminating Vertebrate Host DNA. PLoS ONE.

[37] Horz HP, Scheer S, Huenger F, Vianna ME, Conrads G (2008). Selective isolation of bacterial DNA from human clinical specimens. J Microbiol Methods.

[38] Charalampous T, Alcolea-Medina A, Snell LB, Williams TGS, Batra R, Alder C (2021). Evaluating the potential for respiratory metagenomics to improve treatment of secondary infection and detection of nosocomial transmission on expanded COVID-19 intensive care units. Genome Med.

[39] Piovesan A, Pelleri MC, Antonaros F, Strippoli P, Caracausi M, Vitale L (2019). On the length, weight and GC content of the human genome. BMC Res Notes.

[40] Oxford Nanopore Technologies. 2020. Guppy: Accurate base calling for Oxford Nanopore sequencing. Available from: https://nanoporetech.com/products/guppy

[41] Bachtrog D, Charlesworth B (2001). Towards a complete sequence of the human Y chromosome. Genome Biol.

[42] Li H (2018). Minimap2: Pairwise alignment for nucleotide sequences. Bioinformatics.

[43] Clausen PTLC, Aarestrup FM, Lund O (2018). Rapid and precise alignment of raw reads against redundant databases with KMA. BMC Bioinformatics.

[44] Ombelet S, Barbé B, Affolabi D, Ronat JB, Lompo P, Lunguya O (2019). Best Practices of Blood Cultures in Low- and Middle-Income Countries. Front Med.

[45] O’Grady J, Kay GL, Charalampous T, Aydin A, Scotti R. University of East Anglia. Method for digesting nucleic acid in a sample. Patent WO2021/105659A1. United Kingdom; 2021.

[46] Francis G, Kerem Z, Makkar HPS, Becker K (2002). The biological action of saponins in animal systems : a review. Br J Nutr.

[47] Cooper GM. The Cell: A Molecular Approach. Structure of the Plasma Membrane. Eighth edi. Sunderland, MA, USA: Sinauer Associates (Oxford University Press); 2019.

[48] Brender JR, Mchenry AJ, Ramamoorthy A (2012). Does cholesterol play a role in the bacterial selectivity of antimicrobial peptides?. Front Immunol.

[49] Horvath SE, Daum G (2013). Lipids of mitochondria. Prog Lipid Res.

[50] Casares D, Escribá PV, Rosselló CA (2019). Membrane lipid composition: effect on membrane and organelle structure, function and compartmentalization and therapeutic avenues. Int J Mol Sci.

[51] Ecker DJ, Sampath R, Li H, Massire C, Matthews HE, Toleno D (2010). New technology for rapid molecular diagnosis of bloodstream infections. Expert Rev Mol Diagn.

[52] Ahsanuddin S, Afshinnekoo E, Gandara J, Hakyemezoğlu M, Bezdan D, Minot S (2017). Assessment of REPLI-g multiple displacement whole genome amplification (WGA) techniques for metagenomic applications. J Biomol Tech.

[53] Zheng C, Zhang S, Chen Q, Zhong L, Huang T, Zhang X (2020). Clinical characteristics and risk factors of polymicrobial Staphylococcus aureus bloodstream infections. Antimicrob Resist Infect Control.

[54] Bartlett JG (2004). Nosocomial bloodstream infections in US hospitals: Analysis of 24,179 cases from a prospective nationwide surveillance study. Infect Dis Clin Pract.

[55] Ballantyne KN, van Oorschot RAH, John Mitchell R, Koukoulas I (2006). Molecular crowding increases the amplification success of multiple displacement amplification and short tandem repeat genotyping. Anal Biochem.

[56] Diekema DJ, Hsueh PR, Mendes RE, Pfaller MA, Rolston KV, Sader HS (2019). The microbiology of bloodstream infection: 20-year trends from the SENTRY antimicrobial surveillance program. Antimicrob Agents Chemother.

[57] Gu W, Deng X, Lee M, Sucu YD, Arevalo S, Stryke D (2020). Rapid pathogen detection by metagenomic next-generation sequencing of infected body fluids. Nat Med.

[58] Gosiewski T, Szała L, Pietrzyk A, Brzychczy-Włoch M, Heczko PB, Bulanda M (2014). Comparison of methods for isolation of bacterial and fungal DNA from human blood. Curr Microbiol.

[59] Pinard R, de Winter A, Sarkis GJ, Gerstein MB, Tartaro KR, Plant RN (2006). Assessment of whole genome amplification-induced bias through high-throughput, massively parallel whole genome sequencing. BMC Genomics.

[60] Kai S, Matsuo Y, Nakagawa S, Kryukov K, Matsukawa S, Tanaka H (2019). Rapid bacterial identification by direct PCR amplification of 16S rRNA genes using the MinION™ nanopore sequencer. FEBS Open Bio.

[61] Freed NE, Vlková M, Faisal MB, Silander OK (2021). Rapid and inexpensive whole-genome sequencing of SARS-CoV-2 using 1200 bp tiled amplicons and Oxford Nanopore Rapid Barcoding. Biol Methods Protoc.

[62] Oxford Nanopore Technologies. Rapid sequencing DNA - PCR Barcoding (SQK-RPB004) [Internet]. 2019 [cited 2023 May 8]. Available from: https://community.nanoporetech.com/docs/prepare/library_prep_protocols/rapid-pcr-barcoding/v/rpb_9059_v1_revp_14aug2019

[63] Oxford Nanopore Technologies. Rapid sequencing gDNA - barcoding (SQK-RBK004) [Internet]. 2019 [cited 2023 May 8]. Available from: https://community.nanoporetech.com/docs/prepare/library_prep_protocols/rapid-barcoding-sequencing-sqk-rbk004/v/rbk_9054_v2_revae_14aug2019

[64] Sims D, Sudbery I, Ilott NE, Heger A, Ponting CP (2014). Sequencing depth and coverage: Key considerations in genomic analyses. Nat Rev Genet.

[65] Feldgarden M, Brover V, Haft DH, Prasad AB, Slotta DJ, Tolstoy I (2019). Validating the AMRFINder tool and resistance gene database by using antimicrobial resistance genotype-phenotype correlations in a collection of isolates. Antimicrob Agents Chemother.

[66] Zhao S, Tyson GH, Chen Y, Li C, Mukherjee S, Young S (2016). Whole-genome sequencing analysis accurately predicts antimicrobial resistance phenotypes in Campylobacter spp. Appl Environ Microbiol.

[67] Woodford N, Carattoli A, Karisik E, Underwood A, Ellington MJ, Livermore DM (2009). Complete nucleotide sequences of plasmids pEK204, pEK499, and pEK516, encoding CTX-M enzymes in three major Escherichia coli lineages from the United Kingdom, all belonging to the international O25:H4-ST131 clone. Antimicrob Agents Chemother.

[68] Gekenidis MT, Rigotti S, Hummerjohann J, Walsh F, Drissner D (2020). Long-term persistence of blactx-m-15 in soil and lettuce after introducing extended-spectrum β-lactamase (Esbl)-producing escherichia coli via manure or water. Microorganisms.

[69] Chen S, Larsson M, Robinson RC, Chen SL (2017). Direct and convenient measurement of plasmid stability in lab and clinical isolates of E. coli. Sci Rep..

[70] Dean FB, Nelson JR, Giesler TL, Lasken RS (2001). Rapid amplification of plasmid and phage DNA using Phi29 DNA polymerase and multiply-primed rolling circle amplification. Genome Res.

[71] Malachowa N, Deleo FR (2010). Mobile genetic elements of Staphylococcus aureus. Cell Mol Life Sci.

[72] Flannagan SE, Chow JW, Donabedian SM, Brown WJ, Perri MB, Zervos MJ (2003). Plasmid Content of a Vancomycin-Resistant Enterococcus faecalis Isolate from a Patient also Colonized by Staphylococcus aureus with a VanA Phenotype. Antimicrob Agents Chemother.

[73] Mathers AJ, Peirano G, Pitout JDD (2015). The role of epidemic resistance plasmids and international high- risk clones in the spread of multidrug-resistant Enterobacteriaceae. Clin Microbiol Rev.

[74] Marra AR, Wey SB, Castelo A, Gales AC, Cal RGR, do Carmo Filho JR (2006). Nosocomial bloodstream infections caused by Klebsiella pneumoniae: Impact of extended-spectrum β-lactamase (ESBL) production on clinical outcome in a hospital with high ESBL prevalence. BMC Infect Dis..

[75] Rabaan AA, Eljaaly K, Alhumaid S, Albayat H, Al-Adsani W, Sabour AA (2022). An Overview on Phenotypic and Genotypic Characterisation of Carbapenem-Resistant Enterobacterales. Medicina.

[76] Ramirez MS, Nikolaidis N, Tolmasky ME (2013). Rise and dissemination of aminoglycoside resistance: The aac(6′)-Ib paradigm. Front Microbiol.

[77] Schwarz S, Kehrenberg C, Doublet B, Cloeckaert A (2004). Molecular basis of bacterial resistance to chloramphenicol and florfenicol. FEMS Microbiol Rev.

[78] Williams CT, Musicha P, Feasey NA, Adams ER, Edwards T (2019). ChloS-HRM, a novel assay to identify chloramphenicol-susceptible Escherichia coli and Klebsiella pneumoniae in Malawi. J Antimicrob Chemother.

[79] Falagas ME, Apostolou KE, Pappas VD (2006). Attributable mortality of candidemia: A systematic review of matched cohort and case-control studies. Eur J Clin Microbiol Infect Dis.

[80] Perczyk P, Wójcik A, Broniatowski M (2020). The role of phospholipid composition and ergosterol presence in the adaptation of fungal membranes to harsh environmental conditions – membrane modeling study. BBA - Biomembr.

