# Supplementary material for: Development and proof-of-concept demonstration of a clinical metagenomics method for the rapid detection of bloodstream infection
Source: BMC Med Genomics. 2024 Mar 5;17:71. doi: 10.1186/s12920-024-01835-5 (PMC10916079; doi:10.1186/s12920-024-01835-5)
Supplement: Supplementary file 1 — Supplementary Material 1. [file 12920_2024_1835_MOESM1_ESM.pdf]

Additional file 1: Supplementary Fig. S1 and Tables S1-4

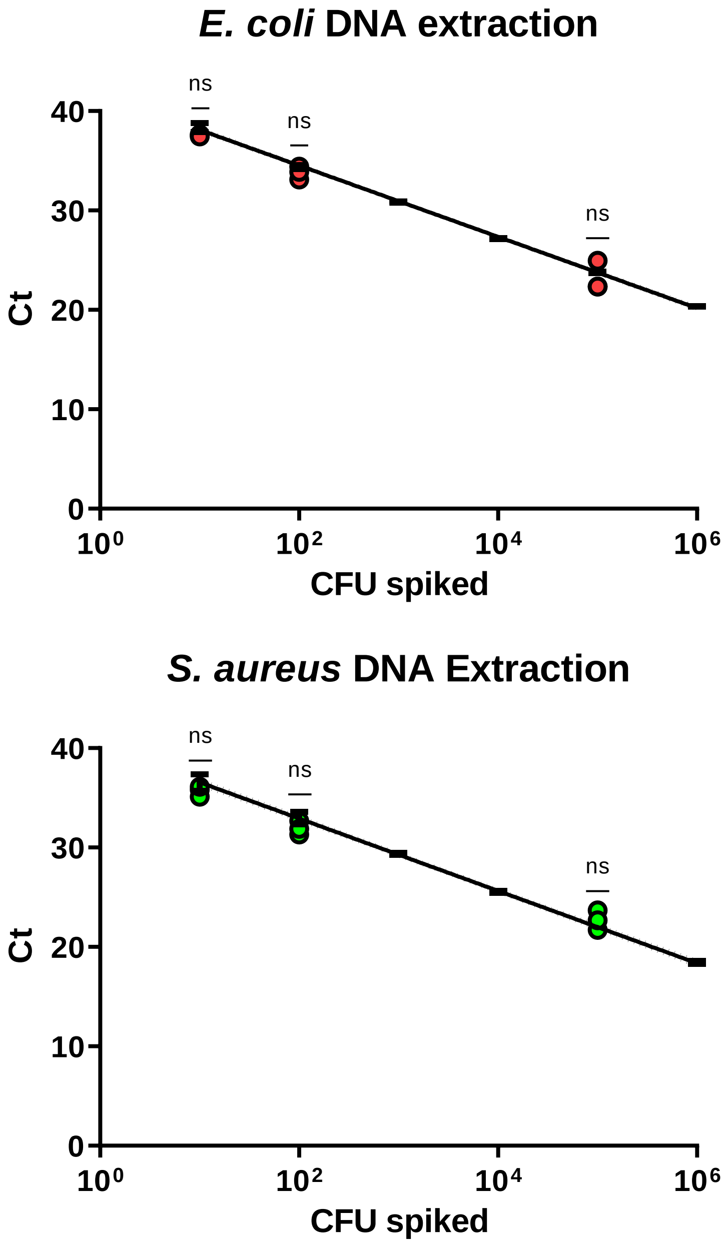

Fig. S1. . E. coli or S. aureus were spiked in blood and underwent DNA extraction protocol. Ct values obtained from blood samples spiked with 10^1, 10^2 or 10^5 of either *E. coli* (top) or *S. aureus* (bottom) and underwent DNA extraction protocol (in dots). The regression lines represent Ct results obtained on 10-fold titrated concentrations of gDNA equivalent to 10^1-10^6 and are added for comparison purposes. Data are individual data points. n = 3 are biological replicates. ns > 0.05, *$p \leq 0.05$, **$p \leq 0.01$. ***$p \leq 0.001$, ****$p \leq 0.0001$.

Table S1. qPCR gene targets and primers sequences used in this project.

Organism	Gene target	Forward primer (5'-3')	Reverse primer (5'-3')	Probe (5'-3')	Reference
Human Chromosome	RNA polymerase A	TGAAGCCGTGC GGAAGG	ACAAGAGAGCC AAGTGTCG	[6FAM]TACCACGT CATCTCCTTTGATG GCTCC TAT[BHQ1]	Charalampous T, Kay GL, Richardson H, Aydin A, Baldan R, Jeanes C, et al. Nanopore metagenomics enables rapid clinical diagnosis of bacterial lower respiratory infection. Nat Biotechnol. 2019;37(7):783–92.
Human Mitochondria	*MT-TL1*	CACCCAAGAAC AGGGTTTGT	TGGCCATGGGT ATGTTGTTA	Sybr Green Master experiment	Weerts MJA, Sieuwerts AM, Smid M, Look MP, Foekens JA, Sleijfer S, et al. Mitochondrial DNA content in breast cancer: Impact on in vitro and in vivo phenotype and patient prognosis. Oncotarget. 2016;7(20):29166–76
E. coli	*cyaA*	CGATAATCGCC AGATGGC	CCTAAGTTGCA GGAGATGG	[6FAM]TAGAGCGC CTTCGGTGTCGGT [BHQ1]	Charalampous T, Kay GL, Richardson H, Aydin A, Baldan R, Jeanes C, et al. Nanopore metagenomics enables rapid clinical diagnosis of bacterial lower respiratory infection. Nat Biotechnol. 2019;37(7):783–92.
S. aureus	*eap*	ACTGTAACTTT GGCACTGG	CAAAGCTTCAA AAGCAGCCTCT A	[6FAM]CTCAAGTT GGAAACCACGAGT AAGAGTGATGAA [BHQ1]	Charalampous T, Kay GL, Richardson H, Aydin A, Baldan R, Jeanes C, et al. Nanopore metagenomics enables rapid clinical diagnosis of bacterial lower respiratory infection. Nat Biotechnol. 2019;37(7):783–92.

Table S2. All species detect at 1mL pipeline 1-5 CFU/mL of *K. pneumoniae* or *E. faecalis* samples.

Limit of detection experiments sample	Species identified (Number of reads/Bacterial population relative abundance)
Klebsiella pneumoniae 1-5 CFU/mL samples	
Replicate 1	*Lactobacillus iners* (3/30%)<br>*Gardnerella vaginalis* (3/30%)<br>*Aerococcus christensenii* (2/20%)<br>*Fannyhessea vaginae* (1/10%)<br>*Gardnerella leopoldii* (1/10%)
Replicate 2	*Serratia marcescens* (388/50.4%)<br>*Cutibacterium acnes* (227/29.5%)<br>*Staphylococcus epidermis* (127/16.5%)<br>*Staphylococcus capitis* (14/1.8%)<br>*Klebsiella pneumoniae* (11/1.4%)<br>*Citrobacter freundii* (3/0.4%)
Replicate 3	*Curibacterium acnes* (11/55%)<br>*Klebsiella pneumoniae* (3/15%)<br>*Ralstonia picketii* (2/10%)<br>*Escherichia coli* (2/10%)<br>*Rhodococcus erythropolis* (1/5%)<br>*Stenotrophomonas maltophilia* (1/5%)
Enterococcus faecalis 1-5 CFU/mL samples	
Replicate 1	*No bacterial reads identified*
Replicate 2	*No bacterial reads identified*
Replicate 3	*No bacterial reads identified*

Listed are all bacterial reads obtained on samples from LoD experiments *'1mL standard protocol'* 1-5 CFU/mL *K. pneumoniae* and 1-5 CFU/mL *E. faecalis.* From all the LoD experiments samples, only in these two occasions the relative abundance of spiked species was ≥10% so we checked for any other potential positive bacteria result. *Number of reads* = total number of reads obtained taxonomically classified as the particular species. *Bacterial population relative abundance* = abundances of species reads over the total population of taxonomically classified reads obtained on the sample.

Table S3. All species detect at NTC samples on 1mL and 5mL CMg pipelines.

'1mL standard protocol'		*'5mL quick-enrichment protocol'*	
Sample	Species identified (Number of reads/Bacterial population relative abundance)	Sample	Species identified (Number of reads/Bacterial population relative abundance)
1	*Cutibacterium acnes* (4/80%)<br>*Corynebacterium kroppenstedtii* (1/20%)	1	*Cutibacterium acnes* (1/100%)
2	*Staphylococcus capitis* (1/100%)	2	*No bacterial reads identified*
3	*Cutibacterium acnes* (19/82.5%)<br>*Neisseria animaloris* (1/3.5%)<br>*Acinetobacter lwoffii* (1/3.5%)<br>*Cutibacterium acnes* (1/3.5%)<br>*Streptococcus mitis* (1/3.5%)<br>*Staphylococcus capitis* (1/3.5%)	3	*No bacterial reads identified*
4	*Cutibacterium acnes* (3/25%)<br>*Cupriavidus metallidurans* (2/17.5%)<br>*Acinetobacter johnsonii* (2/17.5%)<br>*Staphylococcus epidermidis* (1/8%)<br>*Bradyrhizobium sp. CCBAU* (1/8%)<br>*Brucella anthropic* (1/8%)<br>*Sphingobium fuliginis* (1/8%)<br>*Corynebacterium kroppenstedtii* (1/8%)	4	*Klebsiella pneumoniae* (1/100%)
5	*Staphylococcus epidermidis* (3/60%)<br>*Cutibacterium acnes* (2/40%)	5	*No bacterial reads identified*
6	*Cutibacterium acnes* (1/100%)	6	*No bacterial reads identified*
7	*No bacterial reads identified*		
8	*Moraxella osloensis* (2/40%)<br>*Cutibacterium acnes* (1/20%)<br>*Corynebacterium kroppenstedtii* (1/20%)<br>*Malassezia restricta* (1/20%)		
9	Corynebacterium kroppenstedtii (28/20.6%)<br>Paracoccus marcusii (27/19.8%)<br>Pseudomonas aeruginosa (24/17.6%)<br>Streptococcus mitis (23/16.9%)<br>Cutibacterium acnes (18/13.3%)<br>Citrobacter freundii (16/11.8%)		
10	*No bacterial reads identified*		
11	*No bacterial reads identified*		
12	*No bacterial reads identified*		

Table S3. None of the NTC samples on LoD experiments were positive for any bacterial species. Taxonomically classified reads obtained on NTC samples on both *'1mL standard protocol'* and *'5mL quick-enrichment protocol'*. Each limit of detection experiment contained 3 NTC replicate samples, LoD *'5mL quick-enrichment protocol'* experiments with *E. coli* and *S. aureus* shared NTC samples 1-3 and LoD experiments with *K. pneumoniae* and *E. faecalis* shared NTC samples 4-6 as they were performed on the same day. *Number of reads* = total number of reads obtained taxonomically classified as the particular species. *Bacterial population relative abundance* = abundances of species reads over the total population of taxonomically classified reads obtained on the sample.

Table S4. Sequencing metrics on AMR genes using the 5mL CMg pipeline and sequencing for 24 hours.

	Average Coverage (X)			Average template identity (%) / Average Template Coverage (%)		
	5mL protocol					
	50-100 CFU/mL	5-10 CFU/mL	1-5 CFU/mL	50-100 CFU/mL	5-10 CFU/mL	1-5 CFU/mL
blaCTX-M-15	172±45.3	36.5±3.1	46.8±21.7	99.9±0 / 100.0±0	99.9±0 / 100.0±0	99.9±0 / 100.0±0
blaOXA-1	25.0±6.7	7.6±3.2	11.4±6.4	100.0±0 / 100.0±0	100.0±0 / 100.0±0	100.0±0 / 100.0±0
blaTEM-1	11.1±1.3	4.4±1.4	1.4±0.7	99.9±0 / 100.0±0	99.8±0.2 / 101.4±1.4	96.5±3.4 / 101.5±1.2
aac6'-lb-cr	13.0±1.7	3.7±0.6	4.1±2.5	99.8±0 / 100.0±0	99.8±0.1 / 100.6±0.5	99.7±0.1 / 100.5±0.5
mph(A)	121.6±28.6	23.9±2.8	37.2±15.9	100.0±0 / 100.0±0	99.9±0.1 / 100.0±0	100.0±0 / 100.0±0
catB4	15.4±4.5	2.7±0.5	3.7±2.1	91.9±0.9 / 102.4±0.7	91.4±1.3 / 101.7±1.0	94.5±0 / 99.8±0
tet(A)	3.2±0.9	0	1.0±0.6	92.8±7.0 / 96.8±3.2	-	73.2±0 / 77.4±0
dfrA7	16.5±2.6	5.7±0.3	3.5±1.9	99.8±0 / 100.0±0	99.8±0 / 100.0±0	99.3±0.5 / 100.1±0.1
sulI	53.3±11.2	11.6±3.7	10.5±5.2	100.0±0 / 100.0±0	100.0±0 / 100.0±0	99.9±0.1 / 100.1±0.1
aadA5	66.4±17.9	19.3±0.8	14.4±6.3	99.9±0 / 100.0±0	100.0±0 / 100.0±0	100.0±0 / 100.0±0

5mL of blood were spiked with *E. coli* CTX-M-15 (peK499 plasmid) at different concentrations (50-100, 10-50 and 1-5 CFU/mL) and subjected *'5mL quick-enrichment protocol'* with an extended sequencing time (24 hours). *Average Coverage (X)* = average depth of coverage of the template. *Average template identity (%)* = percentage of identical nucleotides between template and consensus. *Average template coverage (%)* = percentage of bases in the template that is covered by consensus sequence. Data are means ± SD. *n* = 3 are biological replicates.
