# Supplementary material for: Development and proof-of-concept demonstration of a clinical metagenomics method for the rapid detection of bloodstream infection
Source: BMC Med Genomics. 2024 Mar 5;17:71. doi: 10.1186/s12920-024-01835-5 (PMC10916079; doi:10.1186/s12920-024-01835-5)
Supplement: Supplementary file 2 — Supplementary Material 2. [file 12920_2024_1835_MOESM2_ESM.pdf]

Additional Fine 2: Supplementary Table 5

Supplementary Table 5. Sequencing data available at ENA.

Sample name	Sample accession	Run accession	Alias
'1mL standard protocol' – 50-100 CFU/mL *E. coli*. R1	ERS16144640	ERR11751810	*1mL_50_100_Ecoli_R1*
'1mL standard protocol' – 50-100 CFU/mL *E. coli*. R2	ERS16144641	ERR11751821	*1mL_50_100_Ecoli_R2*
'1mL standard protocol' – 50-100 CFU/mL *E. coli*. R3	ERS16144642	ERR11751814	*1mL_50_100_Ecoli_R3*
'1mL standard protocol' – 5-10 CFU/mL *E. coli*. R1	ERS16144610	ERR11751781	*1mL_5_10_Ecoli_R1*
'1mL standard protocol' – 5-10 CFU/mL *E. coli*. R2	ERS16144611	ERR11751784	*1mL_5_10_Ecoli_R2*
'1mL standard protocol' – 5-10 CFU/mL *E. coli*. R3	ERS16144612	ERR11751786	*1mL_5_10_Ecoli_R3*
'1mL standard protocol' – 1-5 CFU/mL *E. coli*. R1	ERS16144625	ERR11751794	*1mL_1_5_Ecoli_R1*
'1mL standard protocol' – 1-5 CFU/mL *E. coli*. R2	ERS16144626	ERR11751799	*1mL_1_5_Ecoli_R2*
'1mL standard protocol' – 1-5 CFU/mL *E. coli*. R3	ERS16144627	ERR11751795	*1mL_1_5_Ecoli_R3*
'1mL standard protocol' – 50-100 CFU/mL *S. aureus*. R1	ERS16144649	ERR11751817	*1mL_50_100_Saureus_R1*
'1mL standard protocol' – 50-100 CFU/mL *S. aureus*. R2	ERS16144650	ERR11751816	*1mL_50_100_Saureus_R2*
'1mL standard protocol' – 50-100 CFU/mL *S. aureus*. R3	ERS16144651	ERR11751824	*1mL_50_100_Saureus_R3*
'1mL standard protocol' – 5-10 CFU/mL *S. aureus*. R1	ERS16144619	ERR11751785	*1mL_5_10_Saureus_R1*
'1mL standard protocol' – 5-10 CFU/mL *S. aureus*. R2	ERS16144620	ERR11751788	*1mL_5_10_Saureus_R2*
'1mL standard protocol' – 5-10 CFU/mL *S. aureus*. R3	ERS16144621	ERR11751787	*1mL_5_10_Saureus_R3*
'1mL standard protocol' – 1-5 CFU/mL *S. aureus*. R1	ERS16144634	ERR11751800	*1mL_1_5_Saureus_R1*
'1mL standard protocol' – 1-5 CFU/mL *S. aureus*. R2	ERS16144635	ERR11751804	*1mL_1_5_Saureus_R2*
'1mL standard protocol' – 1-5 CFU/mL *S. aureus*. R3	ERS16144636	ERR11751807	*1mL_1_5_Saureus_R3*
'1mL standard protocol' – 50-100 CFU/mL *K. pneumoniae*. R1	ERS16144646	ERR11751813	*1mL_50_100_Kpneumoniae_R1*
'1mL standard protocol' – 50-100 CFU/mL *K. pneumoniae*. R2	ERS16144647	ERR11751820	*1mL_50_100_Kpneumoniae_R2*
'1mL standard protocol' – 50-100 CFU/mL *K. pneumoniae*. R3	ERS16144648	ERR11751815	*1mL_50_100_Kpneumoniae_R3*
'1mL standard protocol' – 5-10 CFU/mL *K. pneumoniae*. R1	ERS16144616	ERR11751779	*1mL_5_10_Kpneumoniae_R1*
'1mL standard protocol' – 5-10 CFU/mL *K. pneumoniae*. R2	ERS16144617	ERR11751789	*1mL_5_10_Kpneumoniae_R2*
'1mL standard protocol' – 5-10 CFU/mL *K. pneumoniae*. R3	ERS16144618	ERR11751792	*1mL_5_10_Kpneumoniae_R3*
'1mL standard protocol' – 1-5 CFU/mL *K. pneumoniae*. R1	ERS16144631	ERR11751801	*1mL_1_5_Kpneumoniae_R1*
'1mL standard protocol' – 1-5 CFU/mL *K. pneumoniae*. R2	ERS16144632	ERR11751803	*1mL_1_5_Kpneumoniae_R2*
'1mL standard protocol' – 1-5 CFU/mL *K. pneumoniae*. R3	ERS16144633	ERR11751802	*1mL_1_5_Kpneumoniae_R3*
'1mL standard protocol' – 50-100 CFU/mL *E. faecalis*. R1	ERS16144643	ERR11751809	*1mL_50_100_Efaecalis_R1*
'1mL standard protocol' – 50-100 CFU/mL *E. faecalis*. R2	ERS16144644	ERR11751811	*1mL_50_100_Efaecalis_R2*
'1mL standard protocol' – 50-100 CFU/mL *E. faecalis*. R3	ERS16144645	ERR11751812	*1mL_50_100_Efaecalis_R3*

Sample name	Sample accession	Run accession	Alias
'1mL standard protocol' – 5-10 CFU/mL *E. faecalis*. R1	ERS16144613	ERR11751893	*1mL_5_10_Efaecalis_R1*
'1mL standard protocol' – 5-10 CFU/mL *E. faecalis*. R2	ERS16144614	ERR11751780	*1mL_5_10_Efaecalis_R2*
'1mL standard protocol' – 5-10 CFU/mL *E. faecalis*. R3	ERS16144615	ERR11751892	*1mL_5_10_Efaecalis_R3*
'1mL standard protocol' – 1-5 CFU/mL *E. faecalis*. R1	ERS16144628	ERR11751797	*1mL_1_5_Efaecalis_R1*
'1mL standard protocol' – 1-5 CFU/mL *E. faecalis*. R2	ERS16144629	ERR11751796	*1mL_1_5_Efaecalis_R2*
'1mL standard protocol' – 1-5 CFU/mL *E. faecalis*. R3	ERS16144630	ERR11751798	*1mL_1_5_Efaecalis_R3*
'5mL quick-enrichment protocol' – 50-100 CFU/mL *E. coli*. R1	ERS16144706	ERR11751869	*5mL_50_100_Ecoli_R1*
'5mL quick-enrichment protocol' – 50-100 CFU/mL *E. coli*. R2	ERS16144707	ERR11751879	*5mL_50_100_Ecoli_R2*
'5mL quick-enrichment protocol' – 50-100 CFU/mL *E. coli*. R3	ERS16144708	ERR11751884	*5mL_50_100_Ecoli_R3*
'5mL quick-enrichment protocol' – 5-10 CFU/mL *E. coli*. R1	ERS16144670	ERR11751837	*5mL_5_10_Ecoli_R1*
'5mL quick-enrichment protocol' – 5-10 CFU/mL *E. coli*. R2	ERS16144671	ERR11751838	*5mL_5_10_Ecoli_R2*
'5mL quick-enrichment protocol' – 5-10 CFU/mL *E. coli*. R3	ERS16144672	ERR11751833	*5mL_5_10_Ecoli_R3*
'5mL quick-enrichment protocol' – 1-5 CFU/mL *E. coli*. R1	ERS16144688	ERR11751852	*5mL_1_5_Ecoli_R1*
'5mL quick-enrichment protocol' – 1-5 CFU/mL *E. coli*. R2	ERS16144689	ERR11751856	*5mL_1_5_Ecoli_R2*
'5mL quick-enrichment protocol' – 1-5 CFU/mL *E. coli*. R3	ERS16144690	ERR11751855	*5mL_1_5_Ecoli_R3*
'5mL quick-enrichment protocol' – 50-100 CFU/mL *S. aureus*. R1	ERS16144715	ERR11751881	*5mL_50_100_Saureus_R1*
'5mL quick-enrichment protocol' – 50-100 CFU/mL *S. aureus*. R2	ERS16144716	ERR11751883	*5mL_50_100_Saureus_R2*
'5mL quick-enrichment protocol' – 50-100 CFU/mL *S. aureus*. R3	ERS16144717	ERR11751889	*5mL_50_100_Saureus_R3*
'5mL quick-enrichment protocol' – 5-10 CFU/mL *S. aureus*. R1	ERS16144679	ERR11751842	*5mL_5_10_Saureus_R1*
'5mL quick-enrichment protocol' – 5-10 CFU/mL *S. aureus*. R2	ERS16144680	ERR11751843	*5mL_5_10_Saureus_R2*
'5mL quick-enrichment protocol' – 5-10 CFU/mL *S. aureus*. R3	ERS16144681	ERR11751844	*5mL_5_10_Saureus_R3*
'5mL quick-enrichment protocol' – 1-5 CFU/mL *S. aureus*. R1	ERS16144697	ERR11751862	*5mL_1_5_Saureus_R1*
'5mL quick-enrichment protocol' – 1-5 CFU/mL *S. aureus*. R2	ERS16144698	ERR11751863	*5mL_1_5_Saureus_R2*
'5mL quick-enrichment protocol' – 1-5 CFU/mL *S. aureus*. R3	ERS16144699	ERR11751864	*5mL_1_5_Saureus_R3*
'5mL quick-enrichment protocol' – 50-100 CFU/mL *K. pneumoniae*. R1	ERS16144712	ERR11751875	*5mL_50_100_Kpneumoniae_R1*
'5mL quick-enrichment protocol' – 50-100 CFU/mL *K. pneumoniae*. R2	ERS16144713	ERR11751876	*5mL_50_100_Kpneumoniae_R2*
'5mL quick-enrichment protocol' – 50-100 CFU/mL *K. pneumoniae*. R1	ERS16144714	ERR11751874	*5mL_50_100_Kpneumoniae_R3*
'5mL quick-enrichment protocol' – 5-10 CFU/mL *K. pneumoniae*. R1	ERS16144676	ERR11751840	*5mL_5_10_Kpneumoniae_R1*
'5mL quick-enrichment protocol' – 5-10 CFU/mL *K. pneumoniae*. R2	ERS16144677	ERR11751839	*5mL_5_10_Kpneumoniae_R1*
'5mL quick-enrichment protocol' – 5-10 CFU/mL *K. pneumoniae*. R1	ERS16144678	ERR11751841	*5mL_5_10_Kpneumoniae_R1*

Sample name	Sample accession	Run accession	Alias
'5mL quick-enrichment protocol' – 1-5 CFU/mL *K. pneumoniae*. R1	ERS16144694	ERR11751859	*5mL_1_5_Kpneumoniae_R1*
'5mL quick-enrichment protocol' – 1-5 CFU/mL *K. pneumoniae*. R2	ERS16144695	ERR11751858	*5mL_1_5_Kpneumoniae_R2*
'5mL quick-enrichment protocol' – 1-5 CFU/mL *K. pneumoniae*. R1	ERS16144696	ERR11751860	*5mL_1_5_Kpneumoniae_R3*
'5mL quick-enrichment protocol' – 50-100 CFU/mL *E. faecalis*. R1	ERS16144709	ERR11751871	*5mL_50_100_Efaecalis_R1*
'5mL quick-enrichment protocol' – 50-100 CFU/mL *E. faecalis*. R2	ERS16144710	ERR11751872	*5mL_50_100_Efaecalis_R2*
'5mL quick-enrichment protocol' – 50-100 CFU/mL *E. faecalis*. R3	ERS16144711	ERR11751873	*5mL_50_100_Efaecalis_R3*
'5mL quick-enrichment protocol' – 5-10 CFU/mL *E. faecalis*. R1	ERS16144673	ERR11751834	*5mL_5_10_Efaecalis_R1*
'5mL quick-enrichment protocol' – 5-10 CFU/mL *E. faecalis*. R2	ERS16144674	ERR11751835	*5mL_5_10_Efaecalis_R2*
'5mL quick-enrichment protocol' – 5-10 CFU/mL *E. faecalis*. R3	ERS16144675	ERR11751836	*5mL_5_10_Efaecalis_R3*
'5mL quick-enrichment protocol' – 1-5 CFU/mL *E. faecalis*. R1	ERS16144691	ERR11751853	*5mL_1_5_Efaecalis_R1*
'5mL quick-enrichment protocol' – 1-5 CFU/mL *E. faecalis*. R2	ERS16144692	ERR11751854	*5mL_1_5_Efaecalis_R2*
'5mL quick-enrichment protocol' – 1-5 CFU/mL *E. faecalis*. R3	ERS16144693	ERR11751857	*5mL_1_5_Efaecalis_R3*
'1mL standard protocol' – 50-100 CFU/mL *E. coli* CTX-M-15. R1	ERS16144637	ERR11751806	*1mL_50_100_Ecoli_CTXM_R1*
'1mL standard protocol' – 50-100 CFU/mL *E. coli* CTX-M-15. R2	ERS16144638	ERR11751805	*1mL_50_100_Ecoli_CTXM_R2*
'1mL standard protocol' – 50-100 CFU/mL *E. coli* CTX-M-15. R3	ERS16144639	ERR11751808	*1mL_50_100_Ecoli_CTXM_R3*
'1mL standard protocol' – 5-10 CFU/mL *E. coli* CTX-M-15. R1	ERS16144607	ERR11751783	*1mL_5_10_Ecoli_CTXM_R1*
'1mL standard protocol' – 5-10 CFU/mL *E. coli* CTX-M-15. R2	ERS16144608	ERR11751894	*1mL_5_10_Ecoli_CTXM_R2*
'1mL standard protocol' – 5-10 CFU/mL *E. coli* CTX-M-15. R3	ERS16144609	ERR11751782	*1mL_5_10_Ecoli_CTXM_R3*
'1mL standard protocol' – 1-5 CFU/mL *E. coli* CTX-M-15. R1	ERS16144622	ERR11751791	*1mL_1_5_Ecoli_CTXM_R1*
'1mL standard protocol' – 1-5 CFU/mL *E. coli* CTX-M-15. R2	ERS16144623	ERR11751790	*1mL_1_5_Ecoli_CTXM_R2*
'1mL standard protocol' – 1-5 CFU/mL *E. coli* CTX-M-15. R3	ERS16144624	ERR11751793	*1mL_1_5_Ecoli_CTXM_R3*
'5mL quick-enrichment protocol' (3h) – 50-100 CFU/mL *E. coli* CTX-M-15. R1	ERS16144703	ERR11751870	*5mL_50_100_Ecoli_CTXM_3h_ R1*
'5mL quick-enrichment protocol' (3h) – 50-100 CFU/mL *E. coli* CTX-M-15. R2	ERS16144704	ERR11751867	*5mL_50_100_Ecoli_CTXM_3h_ R2*
'5mL quick-enrichment protocol' (3h) – 50-100 CFU/mL *E. coli* CTX-M-15. R3	ERS16144705	ERR11751868	*5mL_50_100_Ecoli_CTXM_3h_ R3*
'5mL quick-enrichment protocol' (3h) – 5-10 CFU/mL *E. coli* CTX-M-15. R1	ERS16144667	ERR11751845	*5mL_5_10_Ecoli_CTXM_3h_R1*
'5mL quick-enrichment protocol' (3h) – 5-10 CFU/mL *E. coli* CTX-M-15. R2	ERS16144668	ERR11751847	*5mL_5_10_Ecoli_CTXM_3h_R1*
'5mL quick-enrichment protocol' (3h) – 5-10 CFU/mL *E. coli* CTX-M-15. R3	ERS16144669	ERR11751848	*5mL_5_10_Ecoli_CTXM_3h_R1*
'5mL quick-enrichment protocol' (3h) – 1-5 CFU/mL *E. coli* CTX-M-15. R1	ERS16144685	ERR11751850	*5mL_1_5_Ecoli_CTXM_3h_R1*
'5mL quick-enrichment protocol' (3h) – 1-5 CFU/mL *E. coli* CTX-M-15. R2	ERS16144686	ERR11751851	*5mL_1_5_Ecoli_CTXM_3h_R2*
'5mL quick-enrichment protocol' (3h) – 1-5 CFU/mL *E. coli* CTX-M-15. R3	ERS16144687	ERR11751849	*5mL_1_5_Ecoli_CTXM_3h_R3*

Sample name	Sample accession	Run accession	Alias
'5mL quick-enrichment protocol' (24h) – 50-100 CFU/mL *E. coli* CTX-M-15. R1	ERS16144700	ERR11751891	*5mL_50_100_Ecoli_CTXM_24h_R1*
'5mL quick-enrichment protocol' (24h) – 50-100 CFU/mL *E. coli* CTX-M-15. R2	ERS16144701	ERR11751877	*5mL_50_100_Ecoli_CTXM_24h_R2*
'5mL quick-enrichment protocol' (24h) – 50-100 CFU/mL *E. coli* CTX-M-15. R3	ERS16144702	ERR11751890	*5mL_50_100_Ecoli_CTXM_24h_R3*
'5mL quick-enrichment protocol' (24h) – 5-10 CFU/mL *E. coli* CTX-M-15. R1	ERS16144664	ERR11751866	*5mL_5_10_Ecoli_CTXM_24h_R1*
'5mL quick-enrichment protocol' (24h) – 5-10 CFU/mL *E. coli* CTX-M-15. R2	ERS16144665	ERR11751878	*5mL_5_10_Ecoli_CTXM_24h_R2*
'5mL quick-enrichment protocol' (24h) – 5-10 CFU/mL *E. coli* CTX-M-15. R3	ERS16144666	ERR11751880	*5mL_5_10_Ecoli_CTXM_24h_R3*
'5mL quick-enrichment protocol' (24h) – 1-5 CFU/mL *E. coli* CTX-M-15. R1	ERS16144682	ERR11751865	*5mL_1_5_Ecoli_CTXM_24h_R1*
'5mL quick-enrichment protocol' (24h) – 1-5 CFU/mL *E. coli* CTX-M-15. R2	ERS16144683	ERR11751861	*5mL_1_5_Ecoli_CTXM_24h_R2*
'5mL quick-enrichment protocol' (24h) – 1-5 CFU/mL *E. coli* CTX-M-15. R3	ERS16144684	ERR11751846	*5mL_1_5_Ecoli_CTXM_24h_R3*
'1mL standard protocol' – NTC. R1	*ERS16144652*	*ERR11751818*	1mL_NTC_R1
'1mL standard protocol' – NTC. R2	ERS16144656	ERR11751825	1mL_NTC_R2
'1mL standard protocol' – NTC. R3	ERS16144657	ERR11751827	1mL_NTC_R3
'1mL standard protocol' – NTC. R4	ERS16144658	ERR11751826	1mL_NTC_R4
'1mL standard protocol' – NTC. R5	ERS16144659	ERR11751828	1mL_NTC_R5
'1mL standard protocol' – NTC. R6	ERS16144660	ERR11751829	1mL_NTC_R6
'1mL standard protocol' – NTC. R7	ERS16144661	ERR11751830	1mL_NTC_R7
'1mL standard protocol' – NTC. R8	ERS16144662	ERR11751832	1mL_NTC_R8
'1mL standard protocol' – NTC. R9	ERS16144663	ERR11751831	1mL_NTC_R9
'1mL standard protocol' – NTC. R10	ERS16144653	ERR11751819	1mL_NTC_R10
'1mL standard protocol' – NTC. R11	ERS16144654	ERR11751822	1mL_NTC_R11
'1mL standard protocol' – NTC. R12	ERS16144655	ERR11751823	1mL_NTC_R12
'5mL quick-enrichment protocol' – NTC. R1	ERS16144718	ERR11751885	5mL_NTC_R1
'5mL quick-enrichment protocol' – NTC. R2	ERS16144719	ERR11751887	5mL_NTC_R2
'5mL quick-enrichment protocol' – NTC. R3	No reads	No reads	*No reads*
'5mL quick-enrichment protocol' – NTC. R4	ERS16144720	ERR11751886	5mL_NTC_R4
'5mL quick-enrichment protocol' – NTC. R5	ERS16144721	ERR11751888	5mL_NTC_R5
'5mL quick-enrichment protocol' – NTC. R6	No reads	No reads	*No reads*
